# Revisiting a decade of inequality in healthcare financial burden in Cambodia, 2009–19: trends, determinants and decomposition

**DOI:** 10.1186/s12939-024-02257-6

**Published:** 2024-09-30

**Authors:** Adélio Fernandes Antunes, Theepakorn Jithitikulchai, Juergen Hohmann, Steffen Flessa

**Affiliations:** 1 Department of Health Care Management, University of Greifswald, Greifswald, Germany; 2 SOCIEUX+ EU Expertise on Social Protection, Labour and Employment, Brussels, Belgium; 3 Faculty of Economics, Thammasat University, Bangkok, Thailand; 4 Takemi Program in International Health, Harvard T.H. Chan School of Public Health, Boston, USA; 5 World Bank Group, Washington, DC USA; 6 General Inspectorate of Social Security, Luxembourg, Luxembourg

**Keywords:** Catastrophic health expenditure, Out-of-pocket health expenditure, Social health protection, Universal health coverage, Financial protection, Financial hardship, Health and inequality, Measurement and analysis of poverty, Sustainable development goals, Cambodia

## Abstract

**Background:**

Out-of-pocket healthcare expenditure (OOPHE) without adequate social protection often translates to inequitable financial burden and utilization of services. Recent publications highlighted Cambodia’s progress towards Universal Health Coverage (UHC) with reduced incidence of catastrophic health expenditure (CHE) and improvements in its distribution. However, departing from standard CHE measurement methods suggests a different storyline on trends and inequality in the country.

**Objective:**

This study revisits the distribution and impact of OOPHE and its financial burden from 2009–19, employing alternative socio-economic and economic shock metrics. It also identifies determinants of the financial burden and evaluates inequality-contributing and -mitigating factors from 2014–19, including coping mechanisms, free healthcare, and OOPHE financing sources.

**Methods:**

Data from the Cambodian Socio-Economic Surveys of 2009, 2014, and 2019 were utilized. An alternative measure to CHE is proposed: Excessive financial burden (EFB). A household was considered under EFB when its OOPHE surpassed 10% or 25% of total consumption, excluding healthcare costs. A polychoric wealth index was used to rank households and measure EFB inequality using the Erreygers Concentration Index. Inequality shifts from 2014–19 were decomposed using the Recentered Influence Function regression followed by the Oaxaca-Blinder method. Determinants of financial burden levels were assessed through zero-inflated ordered logit regression.

**Results:**

Between 2009–19, EFB incidence increased from 10.95% to 17.92% at the 10% threshold, and from 4.41% to 7.29% at the 25% threshold. EFB was systematically concentrated among the poorest households, with inequality sharply rising over time, and nearly a quarter of the poorest households facing EFB at the 10% threshold. The main determinants of financial burden were geographic location, household size, age and education of household head, social health protection coverage, disease prevalence, hospitalization, and coping strategies. Urbanization, biased disease burdens, and preventive care were key in explaining the evolution of inequality.

**Conclusion:**

More efforts are needed to expand social protection, but monitoring those through standard measures such as CHE has masked inequality and the burden of the poor. The financial burden across the population has risen and become more unequal over the past decade despite expansion and improvements in social health protection schemes. Health Equity funds have, to some extent, mitigated inequality over time. However, their slow expansion and the reduced reliance on coping strategies to finance OOPHE could not outbalance inequality.

## Key messages

• The healthcare financial burden and its inequality have sharply increased between 2009–19; disproportionally impacting socio-economically disadvantaged households even with Health Equity Funds offering some mitigation since their national expansion. Urbanization, demographic shifts, and changing disease patterns are key factors in understanding these trends.

• Standard methods for measuring catastrophic healthcare expenditure underestimate the financing burden on less-wealthy households by inflating their consumption and shifting their socio-economic rank.

• The mitigating effect of out-of-pocket spending exemptions and preventive healthcare on both inequality and the overall financial burden of the population suggests a way forward towards UHC for the Cambodian healthcare system.

## Background

### Universal health coverage and equity

Universal Health Coverage (UHC) is encapsulated in the Sustainable Development Goals' Target 3.8. UHC stresses equal access to quality healthcare without financial hardship [1–4]. However, achieving UHC entails budget constraints, forcing governments to prioritize healthcare services, expand coverage, and substitute out-of-pocket spending with prepayment methods [5–7]. UHC embodies equity, efficacy, and efficiency in healthcare use and outcomes [8, 9]. It mandates governments to gradually expand coverage and suitable resource distribution to social sectors based on a country's economic and fiscal capacity [10–12]. Yet, initially expanding coverage can emphasize inequalities. Further challenges like inconsistent benefit packages, administrative procedures, quality healthcare access, transportation expenses, other indirect costs, or qualifying for assistance schemes often arise [6]. Overcoming these hurdles requires open, accountable priority-setting and consistent inequality assessments [7, 9, 13–15].

Measuring equity, or rather equality or inequality, in healthcare financing is a constant endeavor in monitoring UHC. Among the most used indicators of household financial hardship, burden, and economic shocks associated with out-of-pocket healthcare expenditure (OOPHE) are probably impoverishment and catastrophic health expenditure (CHE) [16, 17]. Defining these indicators and their relevance for policymaking has been the source of much argumentation and revision in the last decades’ literature [18–22].

This study contributes to inequality and financial hardship research, deviating from standard approaches to measure financial shocks through CHE and socio-economic ranking. It employs a wealth index ranking and revised consumption aggregate to examine the impact and distribution of OOPHE financial burden across Cambodia’s population, diverging from prior works [23–27]. It integrates new estimates, trend analysis of economic shocks, and OOPHE’s financing sources as coping strategies. A comparison between standard methods and alternative measures of financial burden is also included in this study.

Further, this study includes a determinant analysis of financial burden and its inequality in 2019, investigating factors' contributions to changes in inequality from 2014–19, and evaluating the influence of social health protection coverage. The primary research questions and associated methods are summarized in Appendix Table 5.

The following subsections will introduce the concepts and challenges of financial burden and inequality measurements, outlining the rationale for the authors’ methodological choices. Due to the word count limitations, we only introduce Cambodia's social health protection context. For a review of the Cambodian health system, its challenges, and its evolution, the reader may refer to Kolesar et al. (2022) [28].

### Healthcare-related financial burden

#### Catastrophic healthcare expenditure, a proxy for financial hardship and burden

The World Health Organization (WHO) and World Bank's 2015 UHC Monitoring Report defined impoverishment from healthcare expenditure as households both falling under and already below international poverty lines due to OOPHE [29]. Subsequent iterations of the report adjusted poverty lines for impoverishment [16, 17, 30]. Still, robustly measuring impoverishment is difficult. Fernandes Antunes et al. (2022), for example, found that even a shift of US$0.01 from the international poverty line for Cambodia can lead to a nearly 3% variation in estimates for 2014 [31]. The challenges in defining poverty might explain why healthcare-driven impoverishment was not retained as an SDG indicator. However, CHE was set as an SDG indicator without specific targets [32, 33]. Standard CHE methods facilitate global comparisons, trend monitoring, and gauging public interventions against OOPHE's impact. Still, a consensus on these methods among researchers remains elusive [34, 35].

#### Measuring catastrophic healthcare expenditure

Since Xu et al. [36] seminal work—often dubbed the "WHO Method"—there has been an ongoing debate on how to define CHE [37]. Most discussions focus on establishing thresholds representing economic shocks at the household level.

Current metrics lean towards ability-to-pay or total household wealth indicators [3, 18, 29, 38]. Recent UHC and SDG metrics use CHE thresholds rooted in household consumption, set at 10% and 25% [16]. Regardless of the approach, CHE might not entirely reflect the financial struggles of low-income households, especially those already strained by minimal healthcare costs or avoiding such expenses due to the unaffordability of services [18]. Defining financial burden solely through CHE overlooks the nuances of household finances and spending behaviors [39]. Moreover, CHE misses out on the broader implications of healthcare distress spending, like asset selling, child labor, and missed school days.

The WHO Method determines ability-to-pay (commonly referred to as capacity-to-pay) by deducting an allowance for ‘essential’ food consumption from total consumption and equivalizing household sizes, i.e., accounting for the household members’ age structure. In its application, many researchers favor the equalization factor used by Xu et al. (2003) [37], even when detailed household structure data is available. Furthermore, the WHO Method sets essential food consumption on national medians, overlooking regional variations [40].

As an alternative to capacity-to-pay, the standard definition of total consumption encompasses OOPHE, which spikes with health shocks, skewing households’ wealth and socio-economic ranking, and leading to potential bias in inequality measurements. This explains why CHE incidence using consumption ranking appears higher among ‘wealthier’ households [34, 41]. Concurrently, Sas Trakinsky et al. (2020), in their assessment of financial protection in Burkina Faso, deduced that while CHE detects households with health shocks, it poorly correlates with truly disadvantaged groups [42].

Given these considerations, our analysis departs from CHE. It looks at "financial burden" (FB) by defining "excessive financial burden" (EFB) as metrics of healthcare-related financial shocks by excluding OOPHE from total consumption.

### Measuring inequality

Inequality measures for binary health outcomes, like CHE, fall into two categories: stratified measures and ratios using socio-economic quintiles or geographic markers [43]; and measures of concentration across distribution rankings [44, 45].

#### Socio-economic ranking

Households' socio-economic ranking typically uses total consumption, including OOPHE [46]. Yet, understanding wealth and poverty necessitates looking beyond mere consumption, as in measures like the Human Development [47] and Multi-Dimensional Poverty indices [48–50]. *For a comprehensive review of healthcare inequality measures and equity dimensions, see* Pulok et al. (2020) [51]*.* Another weakness of socio-economic ranking through consumption is that this is sensitive to fluctuations in wealth and does not adequately reflect productive assets or resources that could enable households at the lower end of the wealth spectrum to escape poverty traps or keep better-off households out of poverty. To respond to these challenges, Carter and Barrett (2006) proposed a dynamic-asset-based approach to determine the incidence of poverty traps and persistent poverty [52]. However, asserting which assets display such properties or having surveys that capture those is not trivial.

Complex measures of wealth, such as asset-and-housing-characteristics-based wealth indices, are also commonly used for the socio-economic ranking in concentration analysis [53, 54]. Such indices are popular in inequality analysis of demographic health surveys that lack general consumption data [55, 56]. However, wealth indices are far from universal due to their cross-sectional nature and asset weights varying over time and place. This drove Smits and Steendijk (2015) to propose a consistent international wealth index based on their analysis of 165 surveys across 97 countries [57].

The Filmer-Pritchett Principal Component Analysis (PCA) is widely used with binary variables when building wealth indices [58, 59]. However, Howe et al. (2008) and Poirier et al. (2020) criticize this approach because standard PCA was developed for continuous data simplification, and using binary data leads to skewed scores by favoring variables associated with urban wealth. These shortcomings result in ineffective discrimination between wealth assets in rural areas and a limited demarcation between households at the lower end of the socio-economic ranking (also known as ‘clumping’). [60, 61]. Martel et al. (2021) introduced a polychoric dual-component analysis with ordinal variables to address these weaknesses [62]. The inclusion of the second component intends to reflect the wealth structure in rural areas, as suggested by Ward (2014) [63]. In this study, we adopt this approach.

#### Concentration indices

Interpreting inequality requires mathematical translations reflecting inherently subjective social welfare judgments [45]. It's crucial to grasp these judgments when interpreting inequality measures, especially commonly used ones like concentration indices (CI) and their transformed versions [64–66].

CIs reflect normative judgments of inequality in their extreme values: 0 for perfect equality, -1 when the lowest-ranked socio-economic unit entirely captures the variable of interest, and + 1 when this is held by the highest-ranked [67]. This interpretation is straightforward for continuous variables such as income. However, CIs are more challenging to interpret for health-related variables, among others, because of the possible definition of indicators as shortcomings or gains, their scale, their bounded values, and natural means and limits [68, 69]. Transformations of the CI to accommodate such challenges include the General CI, the Wagstaff CI (WCI), and the Erreygers CI (ECI) [70]. The latter two are widely used for binary variables such as CHE [65, 71–73].

WCI and ECI are often termed normalized or corrected CIs. Both correct for the variable's mean distribution in the population and consider the limits of variables like life expectancy [74]. Debates on the relative advantages of both indices have been intense [69, 75, 76]. While reviewing these indicators, Kjellsson and Gerdtham (2013) posited that neither is superior, as their distinction arises solely from normative judgments. Both indices can appropriately reflect health gains and shortcomings (ill-health) through transfer, mirror, and cardinal invariance properties. In addition, ECI is characterized by the level independence property. The latter means that the index is insensitive to proportional increases in the variable of interest across the middle of the distribution. Discussing these properties goes beyond the purpose of this paper. *For a review of the characteristics of concentration indices, the reader may refer to* Kjellsson and Gerdtham (2013)*.* ECI was used in this study.

#### Decomposing inequality measures

CIs are also valued in statistical analyses for their capability to accommodate regression models, pinpoint determinants of inequality, and facilitate inference and group comparisons [70]. Their decomposition provides insights into factors’ contributions and mitigational effect, revealing both means and coefficients variation distributional impact [43].

The Oaxaca-Blinder decomposition is a prevalent technique, with applications spanning time cohort, socio-economic, and geographic classifications [77–81]. Rahimi and Hashemi Nazari [82] *provide a comprehensive and illustrative guide to this method.* The combination of the Oaxca-Blinder decomposition and the Recentered Influence Functions (RIF) methodology—primarily designed for outlier impact assessment [83]—allows for detailed group-wise inequality measure breakdowns [84, 85]. Heckley et al. (2016) expanded RIF's use in index decompositions of binary variables [86]. Notably, Asif and Akbar (2021) and Asuman et al. (2020) applied these methods to study child nutrition and stunting, respectively [87, 88]. *For a detailed mathematical explanation and example of wages decomposition on gender see* Jithitikulchai (2016) [89]*.* We adopt this combination of methods in this study.

### Cambodia’s context

In Cambodia, the Service Coverage Index increased from 19 in 2000 to a ‘high coverage’ score of 61 in 2019, officially steering the nation towards UHC [90, 91]. This achievement may be partly attributed to the expansion of social health assistance through the Health Equity Fund (HEF). HEF offers free public healthcare and hospital transportation to vulnerable populations. Following a decade of segmented operations by various non-profit organizations, a 2015 government initiative sought to nationalize, consolidate, and expand HEF. More recently, a 2017 scheme reform intended to extend its scope to select informal economy workers [92].

HEF beneficiaries are primarily identified by proxy means testing and community consultation through a national program, IDPoor. In addition, ex-post needs assessments at public hospitals can provide access to HEF benefits for households that can no longer afford services. Such households are supplied with ‘Primary Access Cards’ for identification [93]. While at core IDPoor identification process employs multidimensional poverty measures [94, 95], the prevailing approach to assess the HEF targeting efficacy has been the correlation between its coverage and household consumption ranking [25]. Furthermore, the national representative consumption and living standards surveys do not enable differentiation between pre- and post-identified households.

Concurrently, the National Social Security Fund (NSSF), a mandatory contribution-based social insurance for the formal sector, has broadened its initially limited benefit package. By 2020, it provided effective coverage for 3.3 million individuals, or approximately 19% of households, albeit still excluding dependents [96–99].

Notwithstanding the expansion of HEF and NSSF schemes, high OOPHE and reliance on coping strategies persist, notably among poorer, larger households and rural areas [26, 90]. In 2014, 12% of individuals encountering health issues borrowed money for treatment, escalating to 28% for bills exceeding US$100 [100], and 2.7% of the population resorted to borrowing or selling assets [97].

## Methods

### Data

We use data from the Cambodian Socio-Economic Surveys (CSES) 2009, 2014, and 2019. These are nationally representative surveys with 10,000–12,500 household interviews. The data is available upon request from the Cambodian National Statistics Institute or the World Bank Data Repository.

### Socio-economic status

#### Consumption aggregates

Two distinct total consumption aggregates (EXP) were constructed:

- An "old" aggregate, incorporating OOPHE and education spending, as reported in specific CSES modules.
- A revised aggregate, encompassing durables and rental consumption alongside all items from the "old" aggregate version but excluding OOPHE.

The old aggregate construction is detailed in Fernandes Antunes et al. (2022) [31]. The revised aggregate follows the recommendations of the authors and integrates previously omitted components like rental consumption for dwelling owners and durable goods consumption, but it excludes OOPHE because of EXP’s elasticity to OOPHE. Rental consumption was estimated from reported rental market values for owned residential dwellings or replaced by actual rental expenditure when available. Missing values for rental consumption were estimated from the median in the sampling unit.

CSES records the number and purchase value of 'new' durable goods acquired within 12 months. For items exceeding this age, households estimated the current rental market value for a similar object in their neighborhood. The revised aggregate only includes non-productive durable goods in line with the Cambodian Demographic Health Survey (CDHS) wealth index and the Cambodian Ministry of Planning’s 2019/20 consumption aggregate. Consumption estimates for these items were determined using their quantity, purchase or market value, and adjusted for life expectancy.

Expenditure and consumption variables were converted to monthly Figs. (30.4 days) in current local currency units (Khmer Riel, KHR, or CU) per household or capita. Conversions to current US Dollars (US$) and constant 2011 Purchasing Power Parity units (International Dollar, INT$) employed deflators from the World Development Indicators database^1^ (World Bank, 2022).

#### Wealth index

The wealth index was adapted from the Cambodian National Institute of Statistics approach used in the CDHS analysis [55]. It includes living standard variables and non-productive assets (durable goods). We employed discrete and ordinal variables with polychoric dual-component analysis with the syntax kindly provided and adapted from Martel et al. (2021) [61, 62].

Key living standard elements include lighting, cooking energy, water sources, sanitation facilities used, dwelling characteristics and size, and qualitative items categorized by quality and financial investment [57]. Housing characteristics such as size and number of rooms were adjusted for person equivalents. Water and sanitation source categorization adheres to the Joint Monitoring Program’s Water and Sanitation Ladders [101].

Durable goods were recoded to discrete ordinal variables considering their monthly consumption. Item value was defined at the 25th, 50th, and 75th percentiles as none, low, medium, and high, respectively. Details on the wealth index calculation and composition are provided with the eigenvalues of the component analysis in Appendix Table 6. Spearman ranking tests for the wealth index showed a higher correlation between this and the revised consumption aggregate, rho 0.67, and the old aggregate, rho 0.50.

#### Household equivalent size

Household equivalent sizes were estimated using Eurostat's OECD Modified [Equivalence] Scale [102, 103]. The calculated average equivalized household sizes were significantly lower than those generated with the WHO Method’s equivalent factor (unreported results).

### Main variables of interest

#### Out-of-pocket healthcare expenditure and funding sources

OOPHE was derived from the CSES's health and expenditure section, capturing illness reports, care-seeking, and related costs per individual in surveyed households, including service types, provider options, transport costs, and funding sources such as income, savings, borrowing, asset sales, and advanced production sales. Transportation costs were excluded to prevent duplication with reported household non-food expenditures. Funding sources were queried in decreasing order, allowing up to three responses, with a presumed proportional reduction in amount per source. Appendix Table 7 presents the allocation method between financing sources.

#### Financial burden

FB is defined as the share of OOPHE over EXP. A household was categorized as experiencing ‘excessive FB’ (EFB) if its OOPHE exceeds the 10% (EFB10) or 25% (EFB25) threshold of total consumption, excluding OOPHE.

A household was defined as experiencing CHE when its OOPHE exceeded 40% of its capacity-to-pay (CTP) based on the old aggregate and standard persons equivalences following the WHO Method [24, 26].

The dummy variables can be mathematically expressed as:

$${\text{EFB}}_{ji}=\left\{\begin{array}{c}{1} \, \, {\text{i}}{\text{f}} \, \frac{{OOPHE}_{i}}{{EXP}_{i}}>\frac{{T}_{j}}{100}\\ 0\,if\,otherwise\end{array}\right.$$

where:

• $OOPHE_{i}$ is the out-of-pocket healthcare expenditure of household *i* excluding transportation costs

• $EFB_{ji}$ is the dummy variable for the excessive financial burden of household *i* at threshold *j,* at *j*: 10 and 25.

• $EXP_{i}$ is the total consumption of household *i* excluding OOPHE.

• $T_{j}$ is the threshold set for the EFB at *j*.

$${CHE}_{i}=\left\{\begin{array}{c}{1} \, \, {\text{i}}{\text{f}} \, \frac{{OOPHE}_{i}}{{CTP}_{i}}>0.4\\\,0\,if\,otherwise\end{array}\right.$$

$${CTP}_{i}={THE}_{i}-{SE}_{i}$$

with

$${SE}_{i}={food}_{{{45}^{th}-55}^{th}}\times {eqsize}_{i}$$

and

$${eqsize}_{i}={hhsize}_{i}^{0.56}$$

where:

• $CHE_{i}$ is the dummy variable for catastrophic healthcare expenditure for household *i.*

• $CTP_{i}$ is the capacity-to-pay for household *i*.

• $SE_{i}$ is the subsistence food consumption of household *i* adjusted for household equivalent size.

• $food_{45th-55th}$ is the weighted average food expenditure per capita for households between the 45th and 55th quintiles, ranked by their share of food expenditure over total consumption including OOPHE.

• $eqsize_{i}$ is the person equivalent size of household *i*.

• $hhsize_{i}$ is the unadjusted number of members for household *i*.

• 0.56 is the WHO standard equivalent size adjustment factor.

### Explanatory variables

The analysis incorporates explanatory variables like geographic strata, household structure, head characteristics (age, education, ethnicity, marital status, gender, and disability status), access to water, use of sanitation facilities, social protection coverage, free healthcare utilization, vulnerabilities, healthcare behavior, disease prevalence, OOPHE funding sources, and coping strategies. Appendix Table 8 includes summary statistics for all explanatory variables from 2009–19.

#### Geographic strata

Before 2019, CSES geographic stratification was confined to three regions: Phnom Penh, other urban, and other rural areas. Since then, the categorization was expanded to five zones: Phnom Penh, Plain, Tonle Sap, Coastal, and Plateau and Mountains. In addition, dwellings are categorized as urban and rural. Data from 2009–14 was recoded to accommodate the revised categorization.

#### Access to free healthcare and social protection

CSES tracks access to subsidized healthcare, inquiring about households' utilization of free healthcare in the preceding 12 months and the exemption source, like listing on a poor household roster or insurance. An additional dummy variable for free care was constructed, which defined individuals utilizing services in the past 30 days without paying (zero OOPHE excluding transportation costs). Social protection coverage, through mechanisms like HEF (including Priority Access Cards) and NSSF, was determined based on insurance card ownership.

#### Vulnerabilities, coping strategies, and liabilities

CSES incorporates a section on household vulnerability in the last 30 days and past year. Coping strategies defined by the CSES include changes in food sources, borrowing or asking for help for food, reducing meals, selling household assets, foregoing essential expenditures such as education and health, illegal income activities, economic migration, and begging.

Additional variables were constructed for dropouts within compulsory schooling age, and loans for general and illness-related purposes. These indicators are supplemented by variables on coping strategies, including work cessation due to illness and incapacitation because of hospitalization, and use of non-income funding of OOPHE.

#### Disease prevalence and healthcare-seeking

Healthcare-related needs and consumption variables were constructed from the health section data of CSES for the 30 days prior to the interview. The section is structured into two distinct subsections on needs and consumption, so it is impossible to assert which healthcare need services were sought for a specific reported need. However, households report on their seeking of care when ill, and the impact of illness on their activities. Healthcare-seeking data includes the number of visits per individual, first and last provider type visited and, since 2011, hospitalization and inpatient days. Non-illness-related care needs, including maternity care and preventive services, are captured.

Illnesses for which symptoms are prevalent or treatment sought for more than 12 months were previously considered chronic [24], but we categorized these generically as ‘long illnesses’. The 2019 CSES captured detailed causes of disease for 74 conditions for over 5787 households and 7882 individuals. This data was categorized into communicable-infectious diseases (respiratory diseases; and, other infections), chronic-degenerative diseases (neoplasms; endocrine, metabolic and digestive diseases; circular system diseases; and respiratory chronic diseases), injuries and trauma, and other chronic conditions.

#### Processing of outliers and data cleaning

OOPHE outliers were not excluded. Data was curated for inconsistencies in healthcare and durable goods consumption due to data entry issues, such as omitted zeros, values under KHR1000 for durable goods, duplicate entries, and over-reported items.

### Statistical analysis

#### Means, medians, and differences testing

Analyses were processed using Stata 17 with survey settings or sample weights [104]. Variables’ means were tested through pairwise comparison of linear regression estimates without multiple-comparisons adjustment [105, 106]. For zero-inflated variables, medians and their differences were assessed via quantile regression (Cameron and Trivedi, 2022, chapter 15) [105]. Throughout this paper, “significant” only denotes statistical test results surpassing the 95% threshold (*p* ≤ 0.05). Table 1 includes the means, concentration indices, and results of differences’ testing for key variables of interest.

**Table 1:** Key variables means and concentration indices, annual measures and differences testing for all households

			**Annual measure**			**Differences**		
			**Survey year**			**Survey year**		
**Variable**	**Unit**	**Statistic**	**2009**	**2014**	**2019**	**2014vs2009**	**2019vs2014**	**2019vs2009**
**Excessive financial burden from healthcare (EFB) incidence in the last 30 days**								
At 10% of consumption, excluding out-of-pocket expenditure	Percentage of HHs	Mean	10.95%**	12.77%**	17.92%**	1.83%**	5.15%**	6.97%**
		(Conc. Index)	(-0.027**)	(-0.080**)	(-0.113**)	(-0.053**)	(-0.033*)	(-0.086**)
At 25% of consumption, excluding out-of-pocket expenditure	Percentage of HHs	Mean	4.41%**	5.04%**	7.29%**	0.63%#	2.25%**	2.87%**
		(Conc. Index)	(-0.013**)	(-0.037**)	(-0.062**)	(-0.024**)	(-0.025**)	(-0.049**)
**Consumption in the last month (30.4 days)**								
Consumption excl. out-of-pocket health expenditure (EXP)	INT$(2011)	Mean	755.29**	1,037.73**	1,578.23**	282.44**	540.50**	822.94**
		Median	552.64**	815.90**	1,194.37**	263.27**	378.47**	641.73**
		(Conc. Index)	(0.314**)	(0.275**)	(0.303**)	(-0.039**)	(0.028)	(-0.011)
**Out-of-pocket health expenditure (OOPHE) in the last month (30.4 days)**								
OOPHE as a percentage of EXP	Percent of EXP	Mean	5.07%**	5.91%**	7.67%**	0.84%*	1.76%**	2.60%**
Out-of-pocket health expenditure (OOPHE), excluding transportation	INT$(2011)	Mean	30.49**	52.04**	91.82**	21.55**	39.78**	61.33**
		(Conc. Index)	(0.139**)	(0.071*)	(0.083**)	(-0.068#)	(0.012)	(-0.056)
Out-of-pocket health expenditure (OOPHE), including transportation	INT$(2011)	Mean	34.05**	57.92**	100.29**	23.86**	42.37**	66.23**
		(Conc. Index)	(0.139**)	(0.068*)	(0.085**)	(-0.071*)	(0.016)	(-0.055)
OOPHE by financing source								
Income-financed OOPHE, including transportation	INT$(2011)	Mean		31.41**	66.91**		35.51**	
		(Conc. Index)		(0.119**)	(0.152**)		(0.033)	
Savings-financed OOPHE, including transportation	INT$(2011)	Mean		13.02**	22.77**		9.75**	
		(Conc. Index)		(0.041)	(0.027)		(-0.014)	
Borrowing-financed OOPHE, including transportation	INT$(2011)	Mean		8.91**	6.07**		-2.85	
		(Conc. Index)		(-0.068)	(-0.245**)		(-0.176)	
Selling-assets-and-production-financed OOPHE, including transportation	INT$(2011)	Mean		2.86**	1.88**		-0.98	
		(Conc. Index)		(-0.049)	(-0.031)		(0.017)	
Other-and-unreported-financed OOPHE, including transportation	INT$(2011)	Mean		1.72**	2.65**		0.93	
		(Conc. Index)		(0.239)	(-0.283*)		(-0.523*)	
**Social health protection coverage and free healthcare**								
HEF or Priority Access Card holding	Percentage of HHs	Mean	1.59%**	10.32%**	10.34%**	8.73%**	0.02%	8.75%**
		(Conc. Index)	(-0.027**)	(-0.219**)	(-0.217**)	(-0.192**)	(0.002)	(-0.190**)
Free healthcare in the last 12 months (unspecified)	Percentage of HHs	Mean	4.97%**	8.34%**	9.58%**	3.37%**	1.24%#	4.61%**
		(Conc. Index)	(-0.086**)	(-0.173**)	(-0.103**)	(-0.087**)	(0.070**)	(-0.017)
Free healthcare in the last 12 months (from Health Equity Fund, HEF)	Percentage of HHs	Mean	3.03%**	5.43%**	3.65%**	2.40%**	-1.77%**	0.62%
		(Conc. Index)	(-0.054**)	(-0.114**)	(-0.080**)	(-0.060**)	(0.035**)	(-0.025*)
Free healthcare in the last month (unspecified)	HH members	Mean	0.03**	0.03**	0.05**	0.00	0.01**	0.02**
		(Conc. Index)	(-0.106**)	(-0.273**)	(-0.169**)	(-0.167**)	(0.103#)	(-0.063)
**Liabilities**								
Liabilities (loans), overall, unspecified	INT$(2011)	Mean	627.30**	1,286.95**	6,688.91**	659.64**	5,401.96**	6,061.60**
		(Conc. Index)	(0.253**)	(0.288**)	(0.393**)	(0.035)	(0.105*)	(0.140**)
Liabilities (loans), illness-related	INT$(2011)	Mean	33.00**	55.62**	118.60**	22.61**	62.99*	85.60**
		(Conc. Index)	(-0.084)	(-0.048)	(-0.038)	(0.036)	(0.010)	(0.046)
**Healthcare needs in the last 30 days**								
Illness/injury reported	HH members	Mean	0.69**	0.67**	0.73**	-0.03	0.06**	0.04
		(Conc. Index)	(-0.008)	(-0.040**)	(-0.042**)	(-0.031*)	(-0.003)	(-0.034*)
Long (chronic—> 1 year) illness	HH members	Mean	0.14**	0.13**	0.23**	-0.01	0.10**	0.09**
		(Conc. Index)	(0.041*)	(0.020)	(-0.001)	(-0.021)	(-0.021)	(-0.042#)
Non-illness-related care needs	HH members	Mean	0.18**	0.18**	0.30**	0.00	0.12**	0.11**
		(Conc. Index)	(0.011)	(-0.015)	(0.056**)	(-0.026)	(0.071**)	(0.045)
**Healthcare seeking in the last 30 days**								
Healthcare visits (any provider)	Visits	Mean	1.39**	1.18**	1.19**	-0.21**	0.01	-0.20**
		(Conc. Index)	(-0.002)	(-0.038**)	(-0.022*)	(-0.035#)	(0.016)	(-0.020)
Healthcare for illness/injury sought	HH members	Mean	0.55**	0.65**	0.70**	0.10**	0.05**	0.15**
		(Conc. Index)	(-0.010)	(-0.040**)	(-0.041**)	(-0.030#)	(-0.001)	(-0.032*)
Medical healthcare sought	HH members	Mean	0.52**	0.58**	0.69**	0.06*	0.11**	0.17**
		(Conc. Index)	(0.043**)	(-0.027*)	(-0.026**)	(-0.070**)	(0.002)	(-0.069**)
Hospitalizations	HH members	Mean		0.04**	0.06**		0.02**	
		(Conc. Index)		(-0.072*)	(-0.097**)		(-0.025)	
**Disease impairment in the last 30 days**								
Activity days stopped because of illness/injury	Days	Mean	1.16**	0.80**	0.85**	-0.36**	0.05	-0.31**
		(Conc. Index)	(-0.059*)	(-0.092**)	(-0.139**)	(-0.033)	(-0.048)	(-0.081#)
**Catastrophic health expenditure (CHE) in the last 30 days**								
At 40% of national capacity-to-pay [WHO method]	Percentage of HHs	Mean	5.08%**	4.90%**	7.60%**	-0.18%	2.70%**	2.52%**
		(Conc. Index)	(-0.032**)	(-0.047**)	(-0.075**)	(-0.014#)	(-0.028**)	(-0.043**)

*p*-values at **p ≤ 0.01, *p ≤ 0.05, #*p* ≤ 0.10

#### Determinants of financial burden

EFB determinants were analyzed using zero-inflated logistic (ZIOL) regression [107–109]. Ranked ordinal FB levels (OOPHE/EXP) for the regression outcomes are FB = 0%, 0 < FB < 10%, 10% ≤ FB < 25%, and FB ≥ 25%. These outcomes were chosen as reflecting no (FB = 0%), low (0 < FB < 10%), medium (10% ≤ FB < 25%), and high (FB ≥ 25%) financial burden.

The model, estimated with the Stata command *ziologit*, was considered appropriate to accommodate the inflation of zeros corresponding to the non-users of health services. The zero-inflated and ordered logit components are simultaneously estimated through a single likelihood function. Considering FB levels as ordered categories enables a straightforward interpretation of results. This model is also less sensitive to outliers. An ordered logit regression has the advantage of handling our outcomes as individual equidistant interest as levels with results that can be expressed as odds ratios. The complete model and its results are provided in Table 2. In addition, the model allows for the simultaneous estimation of contrasted predicted probabilities (marginal effects difference) for explanatory variables, results provided in Table 4. The mathematical model and its likelihood estimation equation are briefly introduced below.

$$\text{Pr}\left({Y}_{i}=0\right)=\frac{\text{exp}(Z^{\prime}_{i}{\gamma})}{1+\text{exp}(Z^{\prime}_{i}{\gamma})}$$

$$\text{Pr}(Y_{i}{\leq}{j}\mid{Y_{i}>0})=\frac{\text{exp}(\alpha_{j}-{X}^{\prime}_{i}\beta)}{1+\text{exp}(\alpha_{j}-{X}^{\prime}_{i}\beta)}$$

$$L = \prod\limits^{n}_{i=1} \left[ \underbrace{\left(\frac{\exp (\boldsymbol{Z}^{\prime}_{\boldsymbol{i}} \gamma)}{1 + \exp(\boldsymbol{Z}^{\prime}_{\boldsymbol{i}} \gamma)}\right)^{I(Y_{i}=0)}}_{\text{Zero-inflated component} } \times\underbrace{\left( \left(1 - \frac{\exp(\boldsymbol{Z}^{\prime}_{\boldsymbol{i}} \gamma)}{1 + \exp(\boldsymbol{Z}^{\prime}_{\boldsymbol{i}} \gamma)} \right) \times \prod\limits_{j=1}^{k}\left( \frac{\exp (\alpha_{j} - \boldsymbol{X}^{\prime}_{\boldsymbol{i}}\beta)}{1 + \exp (\alpha_{j} - \boldsymbol{X}^{\prime}_{\boldsymbol{i}}\beta)}\right)^{I(Y_{i} = j)}\right)^{I(Y_{i} > 0)}}_{\text{Ordered logit component}} \right]$$

where:

**Table 2:** Financial burden determinants analysis results using a zero-inflated ordered logit regression on 2019 data only

	**Equation**			
	**Burden level**		**Zero inflation**	
	**Odds ratio (OR)**		**Odds ratio (OR)**	
	**OR [e^Coef.]**	**OR se**	**OR [e^Coef.]**	**OR se**
**Independent variables**				
**Geographic strata**				
Zone (base: 1. Phnom Penh) [dummy]				
Plain	1.747**	(0.348)	0.983	(0.517)
Tonle Sap	1.605*	(0.322)	1.668	(0.844)
Coastal	1.758*	(0.390)	0.987	(0.561)
Plateau/Mountain	1.741**	(0.357)	2.766#	(1.478)
Urban/Rural area = 1, Urban	0.841*	(0.0657)	0.548*	(0.153)
**Socio-economic strata**				
Wealth quintile (base: 1. Poorest) [dummy]				
Second	0.850#	(0.0834)	1.125	(0.395)
Middle	0.717**	(0.0690)	1.148	(0.412)
Fourth	0.581**	(0.0600)	1.026	(0.368)
Wealthiest	0.397**	(0.0480)	4.189**	(1.766)
**Household (HH) structure [dummy]**				
Household size [number of members] (base: 3–4)				
1–2	1.361**	(0.153)	0.970	(0.423)
5–6	0.849#	(0.0726)	1.118	(0.276)
7 and above	0.601**	(0.0646)	2.564**	(0.833)
Other household characteristics				
Fully female household			1.587	(1.057)
Older persons 60 years old and over			0.450*	(0.164)
**HH structure**				
Children under 5 years old [members]			1.502*	(0.270)
Persons with handicaps [dummy]			1.290	(0.504)
**HH head characteristics [dummy]**				
Age group [years old] (base: 35–44)				
17–24			3.718*	(2.043)
25–34			2.930**	(0.930)
45–54			1.214	(0.391)
55–65			1.248	(0.472)
65 and above			0.978	(0.676)
Marital status (base: Married/in cohabitation)				
Divorced/Separated			0.104**	(0.0738)
Widowed			1.467	(0.564)
Never married or in partnership			0.429	(0.399)
Ethnicity (base: Khmer)				
Cham			1.323	(0.976)
Other			0.422	(0.226)
**Social health protection coverage (card holding) [dummy]**				
Health Equity Fund (HEF) or Priority Access Card	0.721**	(0.0859)		
National Social Security Fund (NSSF)	1.020	(0.102)		
**Free healthcare [dummy]**				
HEF free healthcare in the last 12 months			0.270	(0.564)
NSSF free healthcare in the last 12 months			2.885*	(1.362)
Free healthcare excl. transportation in the last month	0.00370**	(0.00101)		
**Vulnerability [dummy]**				
Household members had an accident in the last 12 months			0.835	(0.539)
Handicap prevalent			1.290	(0.504)
**Liabilities**				
Indebted (unspecified reason) [dummy]	1.069	(0.0761)		
**Healthcare needs in the last 30 days**				
Illness or injury [dummy]			252,828**	(317,272)
Long illness prevalent for more than one year [members]	1.235**	(0.0808)		
Diseases [dummy]				
Chronic diseases				
*Neoplasms*	2.301**	(0.596)		
*Circular system diseases*	1.130	(0.145)		
*Other chronic diseases*	0.908	(0.172)		
Infectious diseases				
*Endocrine, metabolic, and digestive diseases*	1.738**	(0.261)		
*Respiratory infections diseases*	0.851	(0.127)		
*Other infections diseases*	1.065	(0.124)		
Injuries/Trauma	1.709*	(0.411)		
Non-illness-related care [dummy]				
Maternal health (Ante- & postnatal care, delivery)	1.084	(0.160)		
Prevention (Vit A, deworming, immunization & health checks)	1.355**	(0.0964)		
**Healthcare seeking in the last 30 days**				
Healthcare sought (any providers) [visits]	1.064**	(0.0197)		
Medical healthcare sought [members]	1.893**	(0.108)		
Inpatient days per hospitalization [days per member]	1.430**	(0.0573)		
**Disease impairment in the last 30 days**				
Activity days lost because of illness [days]	1.038**	(0.00674)		
**Coping strategies**				
Children 6–17 years old out of schooling [dummy]	1.120	(0.115)		
**Out-of-pocket healthcare expenditure (OOPHE) funding sources in the last 30 days [dummy]**				
Savings	1.284**	(0.0880)		
Borrowing	4.710**	(0.929)		
Selling of assets and production	8.400**	(3.941)		
**Constant**			0.00789**	(0.00456)
**/cut1**			0.0447**	(0.0138)
**/cut2**			9.524**	(2.334)
**/cut3**			39.54**	(9.943)
**Observations**	10,075			

*p*-values at **p ≤ 0.01, *p ≤ 0.05, #*p* ≤ 0.10

- ${Y}_{i}$ is the outcome for the household $i$.
- $\text{Pr}\left({Y}_{i}=0\right)$ is the probability of the outcome for the household $i$ is zero (FB = 0%).
- ${\mathbf{Z}}_{\mathbf{i}}$ is a vector of independent variables for the zero-inflated component of the model.
- $\gamma$ is a vector of coefficients for the zero-inflated component.
- $\text{Pr}\left({Y}_{i} \le j|{Y}_{i} >0\right)$ is the probability that the specific outcome for household $i$ is less than or equal to $j$, given that it is greater than zero.
- *j* ∈ { 1,2,3} indexes the non-zero ordered outcomes (0 < FB < 10%, 10% ≤ FB < 25%, and FB ≥ 25%).
- ${\mathbf{X}}_{\mathbf{i}}$ is a vector of independent variables for the ordered logit component.
- ${\alpha }_{j}$ are the cut points for the ordered logit component.
- $\beta$ is a vector of coefficients for the ordered logit component.
- $L$ is the overall likelihood function for the ZIOL regression model.
- $I\left(\bullet \right)$ is an indicator function, which is 1 if the condition inside is true and 0 otherwise

#### Measure of inequality

The [standard] Concentration Index (CI) for continuous variables and Erreygers CI (ECI) for binary variables were estimated and tested using an author-modified version of the Stata command *conindex* to enable the pairwise testing of differences among groups of three [68]. ECI for EFB can be estimated from:

$$ECI=\frac{4\upmu }{1-0}\text{CI}$$

$$CI=\frac{2}{\upmu }{\text{Cov}}\left({\text{EFB}}_{i},R\right)$$

where:

- $ECI$ is the Erreygers Concentration Index.
- $CI$ is the Concentration Index.
- ${\text{EFB}}_{i}$ is the dummy variable for the Excessive Financial Burden for household *i* at a given threshold.
- $R$ is the fractional rank of the household in the socio-economic spectrum.
- ${\text{Cov}}\left({\text{EFB}}_{i},R\right)$ is the covariance of ${\text{EFB}}_{i}$ ranked on $R$.
- $\upmu$ is the mean of EFB at a given threshold among all households.

#### Inequality determinants and decomposition

RIF regression on EFB10 and EFB25’s ECI was applied to the CSES 2019 dataset, using the Stata commands package *rifhdreg* developed by Rios-Avila (2020) [85]. The model is estimated through ordinary least squares regression. Post-estimates of individual RIF values once expressed as a vector allow for the decomposing covariances between groups.

Inequality trends in ECI across years, 2014–19, were decomposed using a two-step method by Firpo et al. (2009, 2018), which combines RIF regression and Oaxaca-Blinder decomposition on post-estimates [83, 84]. Appendix Table 12 and Appendix Table 13 provide the full decomposition model with its independent variables and results on the ECI between 2014 and 2019 for EFB10 and EFB25, respectively.

## Results

### Descriptive statistics

*Descriptive statistics of exploratory variables at the household level by year are provided in* Appendix Table 8. *Subsequent sections only detail statistically significant means and differences, unless noted as stable or constant over time. When unspecified, variations relate to the period 2009–19. Figures are reported as percentages of all households.*

#### Household characteristics

From 2009–19, urban households doubled from 17.97%-37.79%, and Phnom Penh residents grew from 8.90%-14.62%, while Plain zone populations declined from 40.78%-35.26%. Larger households (> 4 members) decreased from 51.94%-42.92%, as did households led by 13–34-year-olds from 24.66%-17.00% and people without formal education from 25.01%-17.92%. Proportions of married/cohabiting and male household heads held steady (~ 79% and ~ 78%). Access to improved water sources and sanitation facilities notably increased from 45.40%-79.73% and 35.86%-80.36%, respectively.

#### Healthcare needs and disease burden

Households experiencing recent illness/injury rose from 44.74%-55.14%. Long-term illness rates held steady at ~ 11.8% between 2009–14 but reached 19.78% by 2019. From 2014–19, infectious diseases also raised from 32.34%-37.85%, with non-respiratory conditions, especially malaria and dengue, becoming the major health concerns in 2019, impacting 33.79% of households. Chronic and cardiovascular diseases affected 19.61% and 11.04% of households, respectively. Awareness and uptake of preventive health needs almost doubled, from 14.49%-23.84% and 10.87%-21.23% between 2014–19, while maternity care needs were reported by 2.73% of households in 2019.

#### Social health protection and healthcare-seeking

Households seeking medical care for illnesses jumped from 35.45%-53.36%, and healthcare visits from 45.27%-56.31%, though per capita visits held at ~ 0.31. The seeking of biomedical professionals grew from 35.29%-52.46% of households, and per capita figures from 11.95%-17.65%. HEF coverage expanded from 1.59%-10.32% between 2009–14 and stabilized thereafter. By 2019, 14.92% of households had a member holding an NSSF card.

Reports of annual free healthcare access doubled from 4.97%-9.58%. Access to free healthcare due to HEF markedly rose from 2.03%-5.43% between 2009–14 but later fell to 3.65%. By comparison, 4.20% accessed free services through NSSF in 2019. Monthly free healthcare access rose from 2.31%-3.82%, albeit the increase was only significant between 2014–19. Free visits per capita grew from 0.0064–0.0119, or 3.10%-4.95% of all visits (data not shown), respectively.

#### Liabilities, vulnerability, and coping strategies

Household indebtedness declined from 37.90%-31.55% between 2009–14, then increased to 34.47%. The average per capita loan soared from INT$352 to INT$4,483. Loans for illness-related reasons decreased from 3.83%-1.66%, but their per capita value among indebted households rose from INT$209 to INT$1,847. School dropouts fell from 12.19%-7.55% (15–17-year-olds) and 3.92%-2.52% (6–14-year-olds).

From 2014–19, reliance on unspecified coping strategies fell from 14.92%-1.98% annually and 3.14%-1.64% monthly. Meanwhile, ~ 15.68% of households consistently used savings for OOPHE, while borrowing for OOPHE reduced from 2.15%-1.62%.

### Financial burden

Appendix Table 9 *provides incidences of EFB by thresholds across years and strata for all households. Appendix Table 8 also includes estimates of CHE using the WHO as used in previous publications by* Jacobs et al. (2016) and Fernandes Antunes et al. (2018) [24, 26].

**Fig. 1:**
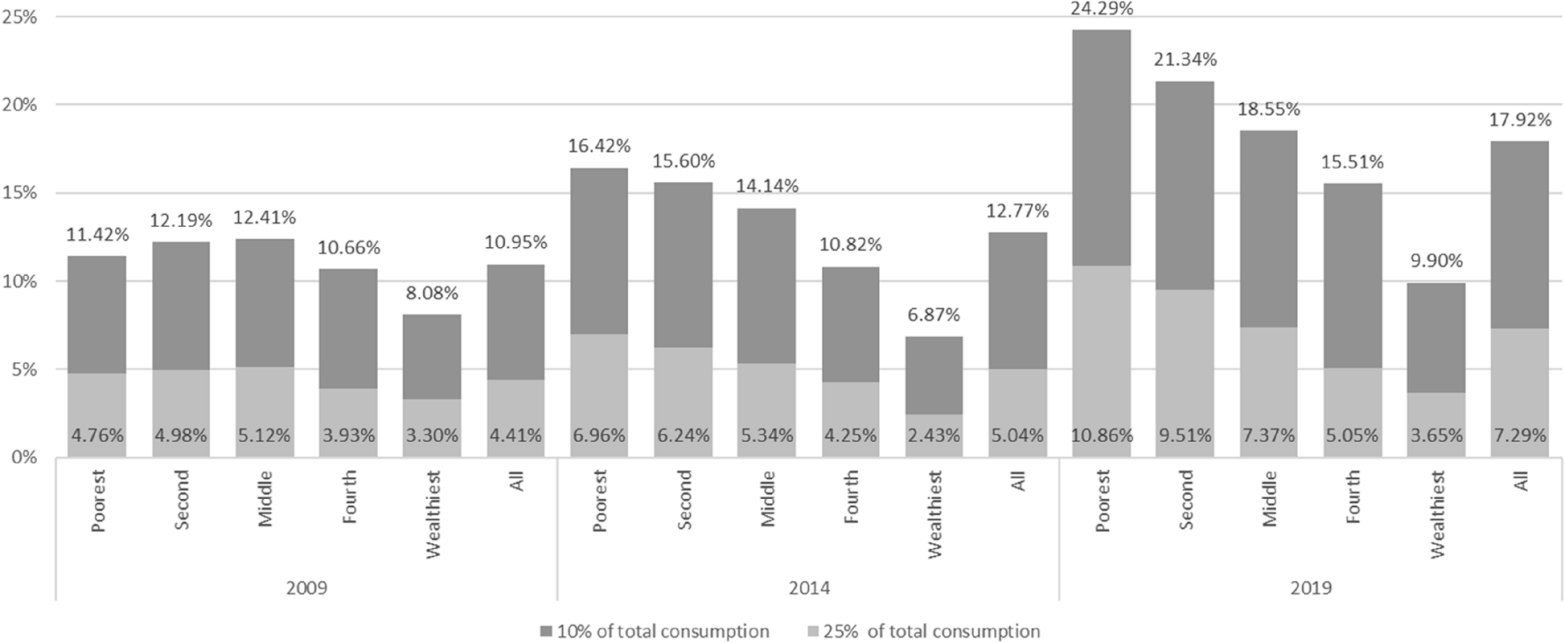
Excessive financial burden incidence among households, defined at 10% and 25% of household’s budget by socio-economic quintile and the national level (all households) [in % of households]. Source: authors calculations

EFB10 and EFB25 incidences at national level and across quintiles for all households are illustrated in Fig. 1. Incidences increased across all categories and years. The uptick was most stark for EFB10, from 10.95%-17.92%, and still rose from 4.41%-7.29% for EFB25. In 2019, EFB10 and EFB25 impacted 24.29% and 10.86% of households in the lowest quintile, respectively. EFB10's rise was not significant for the wealthiest quintile, nor was EFB25's for the wealthiest two quintiles. Appendix Figure 9 illustrates EFB incidences for households reporting healthcare consumption or needs. Among these, the trends were similar to those of the general population. However, national EFB10 and EFB25 in 2019 rose to 31.51% and 12.82%, respectively; for the lowest quintile, these figures peaked at 39.35% and 17.59%.

**Fig. 2:**
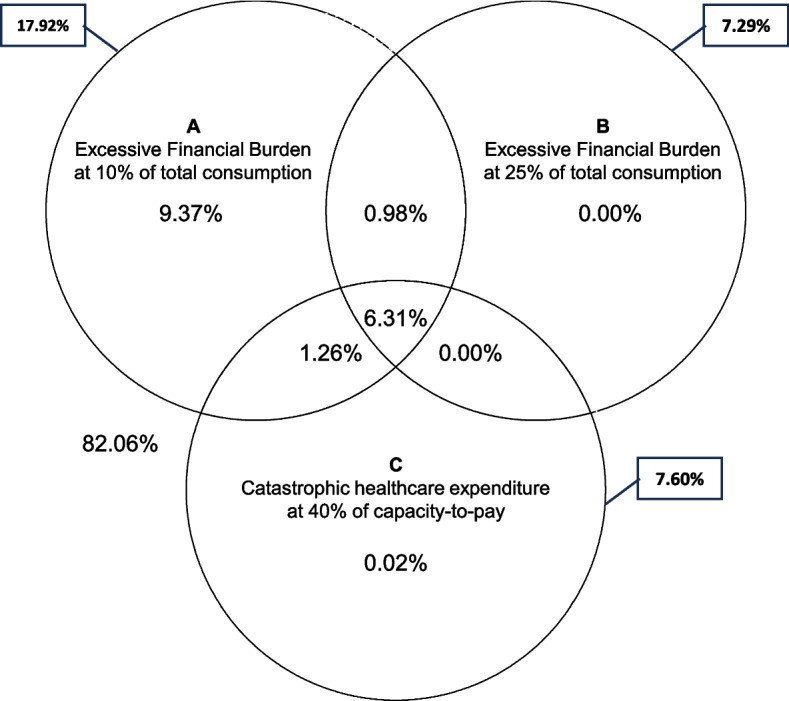
Healthcare-spending-related economic incidence shock estimates among households in 2019, and their overlap using the “World Health Organization Method” on Catastrophic Healthcare Expenditure at 40% of capacity-to-pay, and the Excessive Financial Burden method at 10% and 25% total consumption threshold excluding out-of-pocket healthcare expenditure. Source: authors’ calculations

Figure 2 provides a Venn diagram analysis between the standard WHO Method for CHE at 40% capacity-to-pay, EFB10, and EFB25 in 2019. EFB10 and EFB25 captured over 99% of CHE cases for both years. It is worth noting that this overlap was almost completely captured EFB10 and CHE estimates, suggesting that EFB10 is sufficiently sensitive to capture all economic shocks as defined by the WHO Method. In addition, 0.98% of households were classified as only experiencing EFB25, which would have been missed using CHE.

*The remaining paragraphs present results limited to the incidence of EFB10 in 2019, as EFB25 incidence patterns across strata and years are similar.*

From 2009–19, EFB10 rose across all regions, jumping from 4.30%-7.33% in the capital (Phnom Penh), 12.08%-20.92% in other rural areas, and 7.51%-16.53% in other urban areas. In 2019, EFB10 among fully-female households was 23.20% compared to 17.63% for other households. Incidence was also higher for households with married-under-18-years-old members at 23.04% vs 17.88%. Households with 3–4 members had the lowest incidence.

Households headed by people living with some form of disability had a higher EFB10 of 29.42% vs 16.70%. EFB10 was also higher among households with members living with disabilities, at 29.65% vs 15.53%. EFB10 inversely correlated with the household head's educational attainment, from 23.63% for those devoid of formal education, to 3.99% among those surpassing high school education. Households led by widows/ers had a significantly higher EFB10 incidence at 20.14%, whereas differences across other marital, ethnic, and gender categories were not.

EFB10 was higher among HEF-or-PAC-holding households (across years), at 21.27% vs 17.53%. This counterintuitive pattern was also found for households reporting accessing free healthcare through HEF in the last 12 months, at 22.66% vs 17.74%. However, neither held significance once stratified by wealth quintile. Incidence was lower among households that reported seeking healthcare in the last 30 days without paying, at 7.70% vs 18.32%. Differences across NSSF card holding were not significant. Households with current loans had higher EFB10 incidence. So did households with illness-related loans, at 44.64% vs 17.47%.

Evidently, households reporting any healthcare need or consumption had higher EFB10 incidences. The highest incidence was 57.37% among households with people suffering from neoplasms, and 47.47% for injuries and trauma. When members were hospitalized, this rose to 70.31%.

Households that reported relying on coping strategies in the 12 months prior to the interview or that had 15–17-year-old children dropping out of school also had higher EFB10 incidences.

### Inequality

Table 1* provides the means, CIs, testing results, and medians for key variables on interest by year and absolute differences among all households.* Appendix Table 10 *provides the same table but for the sub-group of households reporting healthcare needs or consumption. For most variables, inequalities were more pronounced in the latter sub-group. However, as the patterns are similar, this section only reviews the results from the general population.*

**Fig. 3:**
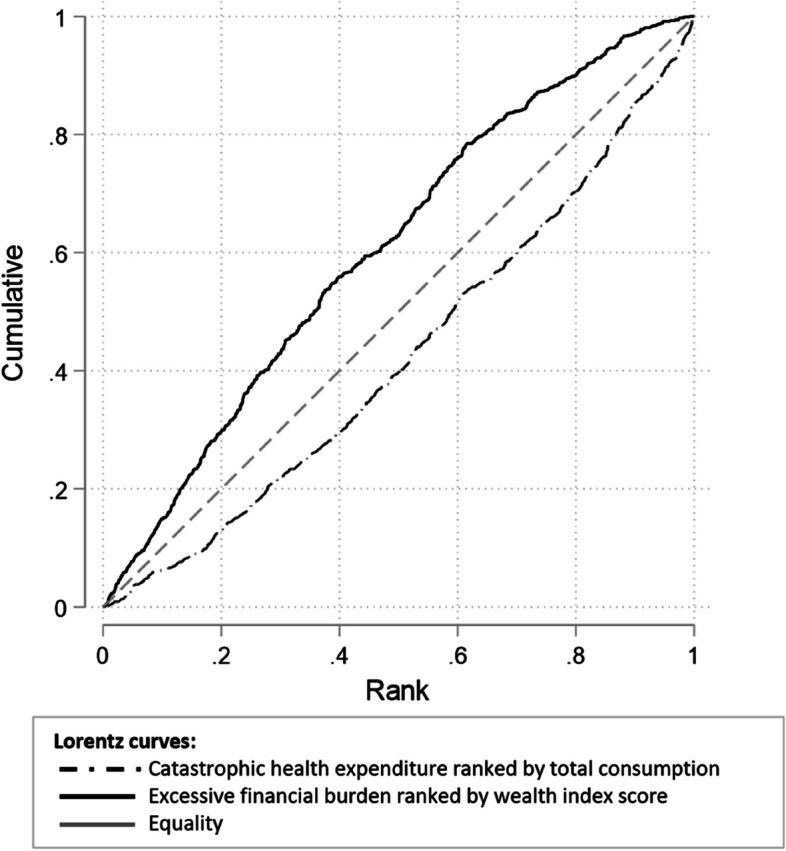
Lorentz concentration curves for Catastrophic Health Expenditure at 40% capacity-to-pay as per the WHO Method at 40% of capacity-to-pay ranked by total consumption, including out-of-pocket healthcare expenditure but excluding rental and durable goods consumptions, and for Excessive Financial Burden at 25% of total consumption ranked by wealth index scores. Source: authors’ calculations

Inequality in EFB10 and EFB25 incidences across households deepened and remained concentrated among the poorest households between 2009–19, from -0.027 to -0.113 for EFB10, and from -0.013 to -0.062 for EFB25. Both EFB and CHE concentrated on the poorest households when using the revised consumption aggregate or wealth index ranking. By contrast, using the old aggregate for ranking and asserting CHE showed a concentration of economic shocks among the wealthy. The behavior of these measures is illustrated with Lorentz concentration curves in Fig. 3.

Over time, the average and the median total consumption, measured by the revised consumption aggregate, more than doubled in constant terms. By 2019, total consumption per household reached INT$1,578 and the median INT$1,194. Between 2009–14, it became more equitable, its CI dropping from 0.314–0.275, but it plateaued afterward.

Monthly OOPHE increased from INT$30.49 to INT$91.82 per household. The average FB, measured as the share of OOPE over total consumption, rose from 5.07%-7.67%. Inequality in OOPHE remained unchanged and concentrated among the wealthier households, reaching 0.083 in 2019. The concentrations were more pronounced in financing sources. Income-finance OOPHE’s CI was 0.152, but for borrowing-financed OOPHE, it was concentrated among the poor at -0.0245. No significant difference was found in the distribution of OOPHE financed from savings and selling of assets.

Liabilities sharply rose, particularly between 2014–19, from INT$1286.95 to INT$6,688.91 for all loans, and from INT$55.62 to INT$118.60 for illness-related loans. No significant inequality was found for illness-related liabilities across all years in contrast to overall loans, which were more concentrated among the wealthier households at 0.393 in 2019.

Significant changes appeared in social health protection coverage and benefits distribution, albeit mainly between 2009–14. HEF (pre-identified households) or Priority Access Card (households post-identified at hospitals) holding was concentrated among the poorest households across all years, reaching -0.217 in 2019. Poorest households benefited more from free healthcare in the 12 months prior to the interviews. However, the distribution remained unchanged between 2009–19, despite an improvement from -0.086 to -0.173 between 2009–19. This V-shape trend in inequality was also found for HEF benefits in the last 12 months.

From 2014–19, inequality in the burden of disease, measured by the number of household members reporting an illness or/and an injury, was significant, at approximately -0.04. Contrastingly, over that period, the distribution of long illnesses was equal. Inequality in the need for non-illness-related care only became significant in 2019.

All healthcare-seeking measures concentrated on households in the lowest part of the socio-economic spectrum from 2014 onwards. By 2019, inequality for healthcare visits was small but pro-poor at -0.022, seeking healthcare for reported illness or injury -0.041, medical healthcare seeking -0.026, and hospitalizations -0.097. Disease impairment, measured by days of activity lost because of illness or injury, tended to disproportionally burden the poorest households over time, with inequality deepening from -0.059 to -0.139 between 2009–19.

### Determinants of the financial burden

Table 2*shows the results of the ZIOL regression on FB expressed in odds ratios for 2019. The table includes two sets of results, one for the zero-inflation and one for the FB levels. The zero-inflated equation results can be interpreted as the likelihood or susceptibility of consuming and spending on healthcare.*

#### Susceptibility to out-of-pocket healthcare expenditure (zero-inflation equation)

A higher susceptibility to healthcare spending was significantly associated with being in the wealthiest quintile versus the poorest (OR 4.189), belonging to a large household of seven or more members compared to 3–4 members (OR 2.564), and residing in a household headed by individuals aged 17–24 years (OR 3.718) or 25–34 years (OR 2.930) versus 35–44 years. Higher susceptibility was also observed in households utilizing NSSF-free healthcare in the past 12 months (OR 2.885) and those reporting a member's illness or injury (OR > 10). Conversely, residing in urban dwellings (OR 0.548), having members aged 60 years or above (OR 0.450), and being led by a divorced or separated head, in contrast to married (OR 0.104), are factors related to a lower susceptibility.

#### Level of financial burden (ordered logit equation)

Households outside Phnom Penh were likelier to have a higher FB (ORs > 1). However, households living in urban areas were less likely to have higher levels (OR 0.841). Those in the three highest quintiles were less likely to experience a higher FB than the poorest households. Compared to households with 3–4 members, smaller households were more likely (ORs > 1), and households with seven or more members were less likely (OR 0.601).

Holding a HEF or PAC card was associated with a lower likelihood of higher financial burden (OR 0.721). However, having at least one household member holding an NSSF card did not significantly influence the odds. Unsurprisingly, having benefited from free healthcare in the last month for at least one household member was associated with a lower likelihood of high levels of FB.

Having members suffering from prolonged illnesses and needing prevention services was associated with an increased likelihood of higher FB levels (OR 1.235). So were neoplasms prevalence (OR 2.301), endocrine, metabolic and digestive infectious diseases (OR 1.738), and injuries/trauma (OR 1.709). Furthermore, the need for preventive services was positively associated with FB levels (OR 1.355). Activity impairment (OR 1.038), seeking healthcare of any sort (OR 1.064), medical healthcare (OR 1.893), and inpatient days per hospitalization (OR 1.430) were associated with higher FB levels.

The association with the financial source of OOPHE was significant and increased from savings (OR 1.284), to borrowing (OR 4.710), to selling of assets (OR 8.400). Having children out of schooling was not significantly associated with a household FB.

#### Individual financial burden levels probabilities (overall model)

Table 3 provides the summary statistics for the four outcomes considered in our ZIOL regression analysis for 2019. Households without OOPHE expenditure (FB = 0%) represented 46.47% of the sample. Households with FB under 10% (0 < FB < 10%) accounted for 35.61%. Of the remaining, 10.63% experienced FB between 10% and under 25% (10% ≤ FB < 25%), and 7.29% had to cope with FB over 25% (25% ≤ FB).

**Table 3:** Financial burden (FB) [out-of-pocket health expenditure as a share of consumption, OOPHE/EXP] statistics for zero-inflated ordered logit regression analysis on 2019 data only

Financial burden (FB) OOPHE/EXP	Frequency	Percentage	Cumulative percentage	Mean	Standard Error	[95% Confidence	interval]
FB = 0%	4,622	45.88	45.88	46.47%	0.76%	44.98%	47.96%
0 < FB < 10%	3,603	35.76	81.64	35.61%	0.69%	34.26%	36.96%
10% ≤ FB < 25%	1,108	11.00	92.64	10.63%	0.35%	9.93%	11.33%
25% ≤ FB	742	7.36	100.00	7.29%	0.33%	6.65%	7.93%
**Total**	10,075	100.00					

*To assess the impact of individual variables with significant effects on the level of FB, we estimated the contrasted predicted probabilities (marginal effects differences) on the entire model at each outcome.* Table 4*provides the estimations by categorical variables. Results for continuous variables are illustrated with charts. In the table and charts, the sum of the probabilities for the four outcomes is one for predictive margins and zero for contrasted predictive margins.*

**Table 4:** Contrasted predicted probabilities (marginal effects difference) for categorical variables with significant effect in Zero-inflated ordered logit regression analysis on 2019 data only

	Financial burden (FB) OOPHE/EXP			
**Outcome**	FB = 0%	0 < FB < 10%	10% ≤ FB < 25%	25% ≤ FB
**Independent variables**				
**Geographic strata**				
Zone (base: 1. Phnom Penh) [dummy]				
Plain	-0.008	-0.043**	0.026**	0.025**
Tonle Sap	-0.012*	-0.031*	0.022*	0.020**
Coastal	-0.008	-0.043*	0.026**	0.025**
Plateau/Mountain	-0.021**	-0.030*	0.027**	0.025**
Urban/Rural area = 1, Urban	0.009**	0.009	-0.009*	-0.009*
**Socio-economic strata**				
Wealth quintile (base: 1. Poorest) [dummy]				
Second	0.001	0.016#	-0.008	-0.009#
Middle	0.003	0.031**	-0.016**	-0.019**
Fourth	0.006*	0.048**	-0.026**	-0.028**
Wealthiest	-0.007	0.093**	-0.043**	-0.043**
**Household (HH) structure [dummy]**				
Household size [number of members] (base: 3–4)				
1–2	-0.003	-0.028*	0.014**	0.018**
5–6	0.001	0.015#	-0.008#	-0.008#
7 and above	-0.006	0.052**	-0.023**	-0.023**
**Social health protection coverage (card holding) [dummy]**				
Health Equity Fund (HEF) or Priority Access Card	0.005*	0.026**	-0.015**	-0.015**
**Free healthcare [dummy]**				
Free healthcare excl. transportation in the last month	0.389**	-0.204**	-0.109**	-0.077**
**Healthcare needs in the last 30 days**				
Diseases [dummy]				
Chronic diseases				
*Neoplasms*	-0.010**	-0.079**	0.036**	0.052**
Infectious diseases				
*Endocrine, metabolic and digestive diseases*	-0.007**	-0.050**	0.026**	0.031**
Injuries/Trauma	-0.007*	-0.049*	0.024*	0.031#
Non-illness-related care [dummy]				
Prevention (Vit A, deworming, immunization & health checks)	-0.004**	-0.026**	0.014**	0.016**
**Out-of-pocket healthcare expenditure (OOPHE) funding sources in the last 30 days [dummy]**				
Savings	-0.003**	-0.021**	0.012**	0.013**
Borrowing	-0.016**	-0.153**	0.053**	0.116**
Selling of assets and production	-0.020**	-0.205**	0.046**	0.179**

*p*-values at **p ≤ 0.01, *p ≤ 0.05, #*p* ≤ 0.10

Households living outside Phnom Penh and in rural areas had significantly higher probabilities of experiencing FB above 10% (10% ≤ FB < 25% and 25% ≤ FB). Living in rural dwellings also significantly reduced the probability of no FB, but it did not affect the probability of FB under 10%.

Compared to the first quintile, households in the three wealthiest quintiles were significantly more likely to experience FB under 10% and less likely to have to cope with FB over 10%. Significant differences in probabilities for no FB were only found with the fourth quintile. No differences in the probability of outcomes were found with the second quintile.

No significant differences were found between the reference households (3–4 members) and those with 5–6 members, or across household sizes on the probability of no FB. However, smaller households (≤ 2 members) were less likely to have FB under 10% and more likely to have FB over 10%. The pattern was inverted for larger households (≥ 7 members).

**Fig. 4:**
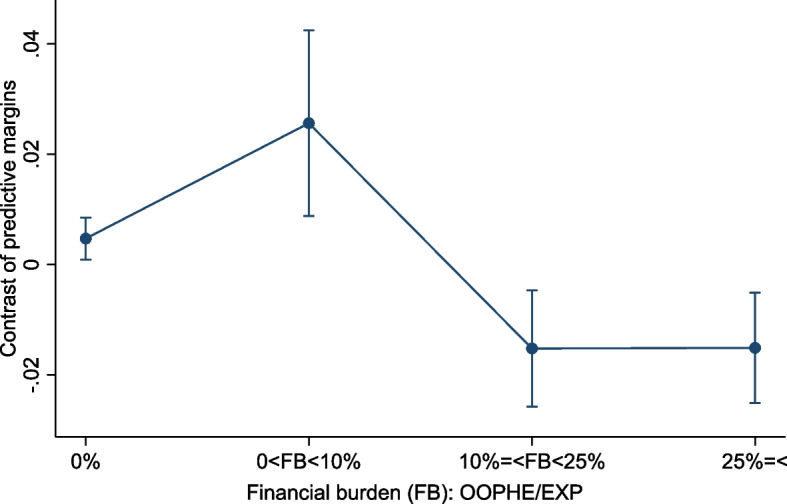
Probability differences (predictive margins contrast) of financial burden outcomes at 0%, 0% to 10%, 10% to 25%, and over 25% of out-of-pocket healthcare expenditure over household consumption between households with a Health Equity Fund or Priority Access Card vs non-holders, in 2019. ﻿Error bars for 95% confidence interval. Source: authors calculations

HEF households were more likely to have no and under 10% FB, and less likely above the 10% threshold. Figure 4 illustrates results for HEF households in different outcomes. As could be expected, households benefiting from free healthcare had significantly higher probabilities of no FB and lower FB across all outcomes.

Prevalence of neoplasms or endocrine, metabolic, and digestive diseases, the need for non-illness-related healthcare significantly lessened the likelihood of no and under 10% FB, and increased the probability of outcomes above 10%. Similar patterns were seen with reported preventive needs and injuries or trauma, though the latter does not significantly impact the probability of FB above 25%.

When relying on OOPHE funding via savings, borrowing, or asset and production sales, households were significantly less likely to have no or under 10% FB, and more likely to exceed the 10% and 25% thresholds.

**Fig. 5:**
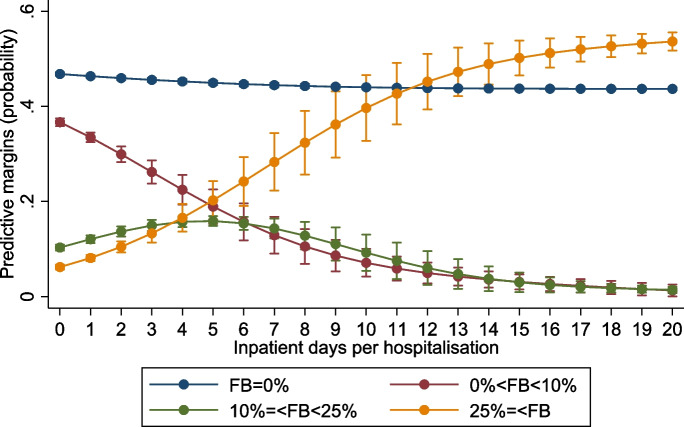
Probability (predictive margins) of financial burden outcomes at 0%, 0% to 10%, 10% to 25%, and over 25% of out-of-pocket healthcare expenditure over household consumption by inpatient days per hospitalization and household, in 2019. ﻿Error bars for 95% confidence interval. Source: authors calculations

Figure 5 illustrates the probabilities (predictive margins) for the four outcomes against inpatient days per hospitalization. Under four days, the most likely outcomes were for a household to have no or FB under 10%. Above five days, households were still more likely to experience no FB, but the probability of EFB25 rapidly rose with hospitalization days and was more likely than the two other outcomes (0% < FB < 10% and 10% =  < FB < 25%). Above 15 days, the most likely outcome was to experience FB above 25%, i.e. EFB25.

**Fig. 6﻿:**
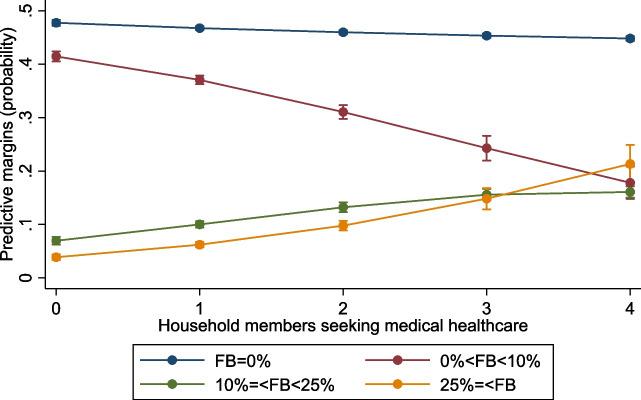
Probability (predictive margins) of financial burden outcomes at 0%, 0% to 10%, 10% to 25%, and over 25% of out-of-pocket healthcare expenditure over household consumption by household members seeking medical healthcare, in 2019. Error bars for 95% confidence interval. Source: authors calculations

Figure 6 illustrates how having household members seeking medical healthcare increases the probability of higher FB outcomes. With three members seeking medical healthcare, the most likely outcomes were that a household would experience no or FB under 10%. However, from 4 members seeking care upwards, a household’s most probable outcomes were no or FB above 25%.

### Decomposition of inequality variation between 2014–19

*In complement to the decomposition analysis results below, the reader will find in the Appendix results from the determinants analysis of EFB inequality for 2019 using RIF regression on ECI of EFB10 and EFB25. These results guided the construction of our Oaxaca-Blinder decomposition model. The results of both analyses are consistent.*

*Appendix Table 12 and Appendix Table 13 provide the results of the Oaxaca-Blinder decomposition on RIF of the ECI differences between 2014-19 for EFB10 and EFB25, respectively. Results are segmented into endowments for means (‘explained’), and effects and interactions (‘unexplained’). Overall (‘total’) results from combined explained and unexplained contributions.*

*The tables display the means for independent variables, coefficients (effects), testing results, and the contribution to total ECI variation by factor [% diff]. The latter are provided in brackets in the remaining paragraphs. We deem the overall contributions as significant only if both the explained and unexplained contributions are also significant.*

**Fig. 7:**
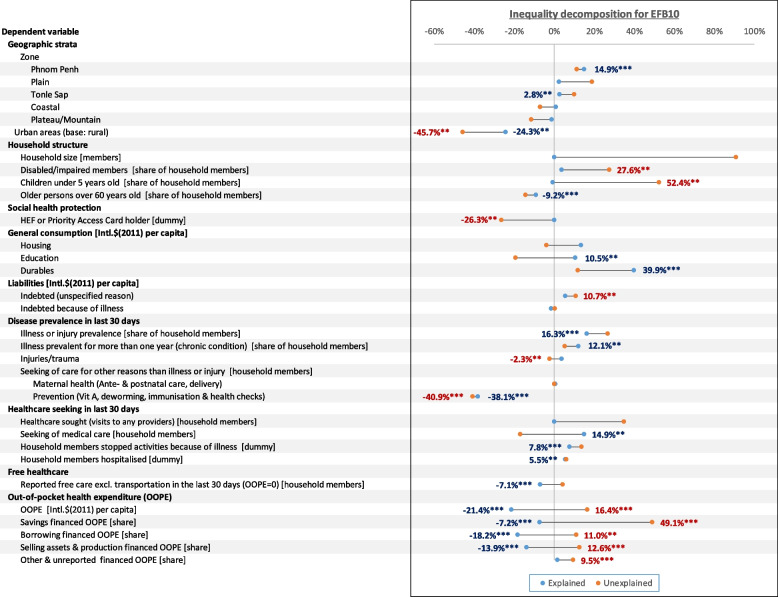
Explained (endowments) and unexplained (coefficients and interactions) results from Oaxaca-Blinder decomposition inequality (Erreygers Concentration Index) increase in excessive financial burden at 10% threshold between 2014–19. Data labels are provided only for results with *p*-values < 0.05 (***p ≤ 0.01, **p ≤ 0.05). Source: authors calculations

**Fig. 8:**
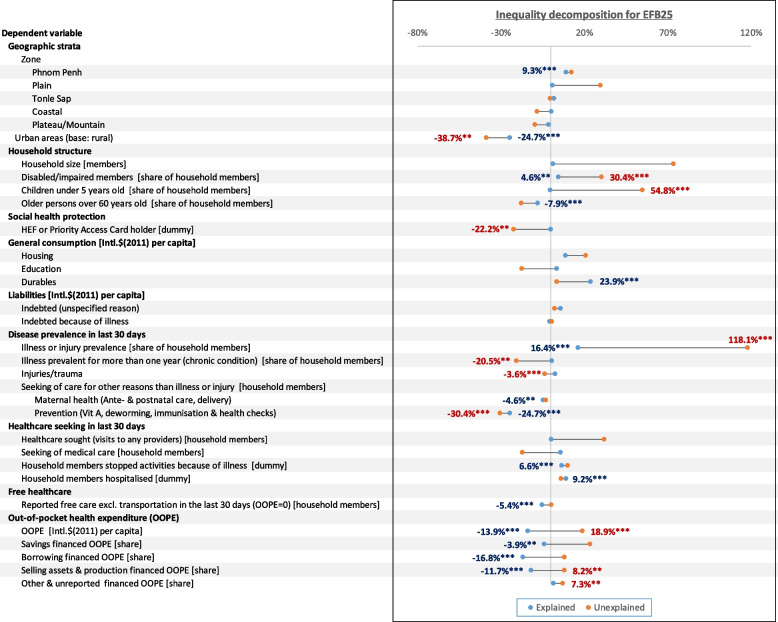
Explained (endowments) and unexplained (coefficients and interactions) results from Oaxaca-Blinder decomposition of inequality (Erreygers Concentration Index) increase in excessive financial burden at 25% threshold between 2014–19. Data labels are provided only for results with *p*-values < 0.05 (***p ≤ 0.01, **p ≤ 0.05). Source: authors calculations

Figures 7 and 8 illustrate the results of the decomposition analysis on inequality for EFB10 and EFB25, respectively. The figures only include data labels for significant results (*p*-values ≤ 0.05).

From 2014–19, EFB inequality shifted further in disfavor of households in the lower half of the wealth spectrum. The ECI for EFB10 fell by 41.05%, from -0.0799 to -0.1130, while EFB25’s ECI worsened by 67.03% from -0.0370 to -0.0618. Most of these can be attributed to unexplained variations in effects and interactions from a few factors.

Overall variations (mean and effect variations) in urbanization had a significantly worsening contribution to EFB10 (-70.06%) and EFB25 inequality (-63.35%). Population ageing, or rather the increase in means of the share of older people in households, mitigated EFB10 (-9.21%) and EFB25 inequality (-7.90%). Conversely, the means variations in households with children under five didn’t significantly alter inequality. However, variations in effects significantly exacerbated EF10 (52.44%) and EFB25 inequality (54.84%). Similarly, variations in effects for the share of household members living with disabilities significantly contributed to EFB10 (30.44%) and EFB25 inequality (27.56%).

Despite no variation in HEF coverage, improvements in its effects notably lessened inequality for both EFB10 (-26.31%) and EFB25 (-22.22%). Concurrently, the increase in mean free healthcare explained a smaller but significant mitigation of EFB10 (-7.13%) and EFB25 (-5.36%).

The jump in consumption of durable goods explained part of the increase in EFB10 (39.94%) and EFB25 inequality (23.87%). Higher education expenditure only significantly explained the change in EFB10 inequality (10.52%).

The variations in the prevalence of illness and injuries among household members had the highest overall contribution to EFB25 (134.52%), particularly in their effects (118.15%). However, only variations in means significantly worsened EFB10 inequality (16.31%). The increased share of household members suffering from long illnesses explained a worsening EFB10 inequality (12.13%). However, the changes in effect for the factor mitigated EFB25 inequality (-20.48%).

Of non-illness-related healthcare needs and utilization subcategories, only preventive services contributed overall to mitigating EFB10 inequity (-78.96%) and EFB25 (-55.04%). These were among the largest in the decomposition. In comparison, and despite a substantial rise, maternity care had a small mitigative contribution to EFB25 inequality (-4.64%) only.

In general, healthcare seeking had no significant contributions to changes in EFB inequality, except for medical healthcare seeking explaining a worsening in EFB10 inequality (14.88%). Having household members stopping regular activities also explained the worsening in EFB10 (7.77%) and EFB25 inequality (6.57%). Similarly, having household members hospitalized members contributed to a further deterioration of EFB10 (5.46%) for EFB10 inequality (9.15%).

OOPHE and OOPHE funding sources' contributions to inequality were mixed. Variations in means mitigated inequality, but variations in effects counterbalanced the latter. Overall, the increase and change in effect in OOPHE mitigated EFB10 inequality (-4.94%) and worsened EFB25 inequality (5.04%). For savings-financed OOPHE, this contributed to an overall worsening in EFB10 (41.89%) inequality. In contrast, overall changes in borrowing-financed OOPE mitigated EFB10 inequality (-7.13%). Similarly, overall changes in OOPHE financed through selling assets and production mitigated EFB10 (-1.28%) and EFB35 inequality (-3.47%).

## Discussion

This study delves in-depth into the evolution, determinants, and inequality of FB in Cambodia over a decade. It departs from the standard definition of healthcare expenditure financial shocks, CHE, by adopting an ‘excessive financial burden’ measurement that separates OOPHE from the total household consumption, and wealth socio-economic ranking approach to asserting inequalities. Our findings diverge from previous and recent conclusions from publications using the standard CHE and consumption aggregates, which found a financial burden in middle consumption quintiles of the population and somewhat positive time trends [24, 25, 27, 110].

Our results suggest that while more stringent, EFB25 can identify instances of the financial burden that CHE misses (Fig. 2). These also indicate that EFB25 helps identify more severe cases of financial burden that the CHE measure may not detect. The results suggest that the EFB measures are more sensitive in asserting FB than standard CHE. EFB10, in particular, appears to be a comprehensive measure, capturing a wide range of economic shocks, including those identified by CHE and additional cases.

In addition to more effectively capturing economic shocks, the revised methods showed a fairer representation of inequality, as shown by contrasting the Lorentz concentration curves for CHE and EFB25 (Fig. 3). The difference in behavior can mainly be attributed to excluding OOPHE from the denominator in EFB and using our wealth index as an alternative to total consumption for socio-economic ranking.

### Evolution of healthcare-related financial burden (2009–19) and distribution

The past decade saw a striking rise in FB nationally, especially amongst the poorest households, accentuating a growing disparity in healthcare affordability. Nearly a quarter of all households in the lowest quintile faced EFB10, and one in 10 experienced EFB25 by 2019. The doubling in incidences among the two lowest quintiles is noteworthy, contrasting with the non-significant rise in EFB10 for the wealthiest quintile.

Geographically, urban areas and regions like Phnom Penh faced lower burdens than rural areas, suggesting that location plays a significant role in determining healthcare expenditures. EFB10 incidence more than doubled in urban areas outside Phnom Penh, and almost tripled for EFB25. More than a fifth of households in rural areas experienced EFB10 in 2019.

By 2019, EFB25 incidence impacted one in ten fully-female households and one in eight households with disabled or handicapped members. Almost a third of households with healthcare needs or consumption experienced EFB10, and one in eight EFB25. Among households with members suffering from long diseases, the figures rose to a staggering two-fifths for EFB10.

The observations align with global patterns, where urban–rural disparities in healthcare access and affordability pervade, often attributed to disparities in infrastructure, income, and health policies [81, 111–116]. For example, Jiang et al. (2019) illustrated that despite over 95% of China's population having public medical insurance, significant disparities in healthcare service utilization and OOPHE across varied income groups persist, especially revealing more healthcare needs and CHE risks among rural residents [117].

### Inequality over time

Reflecting the trends in FB, inequality in the distribution of EFB worryingly increased over time. Alarming is that this trend is uncoupled from the inequality in household consumption, overall healthcare visits, or hospitalizations, which did not significantly change over the decade. The available data does not enable us to account for the quality of services or the type of provider sought; most likely, the inequalities in these are substantial. Inequalities were markedly higher among households with healthcare consumption or needs, but patterns remained similar to those of the general population.

From 2009–19, despite a tripling of OOPHE, both in constant terms and as a portion of total consumption, OOPHE inequality remained unchanged across the population and those consuming or requiring healthcare. However, from 2014–19, income-financed OOPHE leaned towards wealthier households, whereas borrowing-financed OOPHE prevailed among poorer ones, with borrowing emerging as a predominant EFB coping strategy. Concurrently, average household debt over 2009–19 more than decupled and became pro-wealthy, whereas illness-related debt quadrupled but remained equitable. This may be related to a surge in micro-financing access over the past decade [97, 100, 118].

Over the studied period, healthcare-seeking indicators shifted from pro-wealthy to more nuanced, with illness-related metrics like illness/injury incidence, healthcare provider visits, hospitalizations, and lost productivity days increasingly concentrated among the poor by 2019. Within the subgroup needing or consuming healthcare, inequalities lessened yet shifted towards wealthier households, notably in non-illness-related care needs such as maternity care and preventive services.

The concentration of HEF coverage and free healthcare among less wealthy households is a positive finding, and suggests only a limited misallocation of HEF cards, contrary to previous evidence [25]. However, as HEF coverage reported in the CSES did not significantly change between 2014–19, contrary to what would have been expected from official figures. Thus, it seems critical to look at overall exemptions from OOPHE, allocation of HEF benefits, and their distribution.

Across 2009–19, overall use of free healthcare in the preceding 12 months and 30 days significantly increased, barring exemptions via local poor lists. Although distribution remained pro-poor, especially between 2009–14, inequality in these variables diminished from 2014–19. Nevertheless, the average exemptions per household and the percentage of households spared from OOPHE when seeking care in the preceding 30 days increased.

Despite certain positive trends, concerns arise regarding equity from the distribution of FB, debt, and constrained access to exemptions and social health protection coverage. EFB has increasingly burdened the poorest households over time. Notably, only a small portion of the 40% of the population at the lower end of the socio-economic spectrum benefited from exemptions. This persistent inequity and resultant population segregation potentially threaten the social cohesion essential for fair-sustainable socio-economic development [119].

### Determinants of financial burden

The zero-inflated model highlighted several variables significantly associated with households’ likelihood to incur OOPHE and FB levels after adjusting for covariates. Larger, wealthier households, those with heads under 35, and those with children under five using NSSF-paid healthcare in the last year were particularly prone to OOPHE spending. Conversely, households in urban areas, those with members above 60, and those led by divorced or separated individuals exhibited reduced susceptibility.

Although households outside Phnom Penh were less likely to avoid spending or maintain FB under 10%, they were likelier to experience EFB at both thresholds. Urban areas were generally more likely not to spend on OOPHE but less likely to experience FB above 10%. No significant differences were found between the first two quintiles regarding likely FB levels. In comparison, the three wealthiest quintiles tended to maintain FB under 10% and were less likely to exceed this mark, a trend mirrored in rural areas. This, alongside significantly lower illness or injury prevalence in the three wealthiest quintiles, implies potential disparities in accessing expensive healthcare, presumably more qualitative.

Some health conditions significantly drive FB levels. The prevalence of neoplasms, endocrine, metabolic and digestive diseases, and injuries correlate with a higher likelihood of FB above 10%. These outcomes are consistent with existing literature discussing the financial toxicity of cancer [120–122]. The unanticipated positive association between high levels of FB and preventive services, which may encompass costly and capital-intensive elective health checks, warrants further research.

As anticipated, the number of household members hospitalized and the duration of hospitalization are significantly associated with FB levels. Households tend to encounter EFB25 beyond five inpatient days per hospitalization and when over three members sought medical healthcare in the past 30 days. Kastor and Mohanty [123] found comparable outcomes in India, where hospitalization for cancer was the most common diagnosis associated with CHE (79%).

### Coping and financing strategies

Our findings show a strong association between EFB and income-alternative sources of financing for OOPHE, such as savings, borrowing, and selling assets. While the proportion of households using savings remained stable from 2014–19, the relative share of savings, borrowing, and selling assets in financing OOPHE declined, even as the share of households relying on them persisted. The incidence of loans specifically for illness costs halved from 2009–19. Nevertheless, a significant increase was observed in reliance on borrowing and asset sales for those encountering EFB between 2014–19.

No statistically significant relationship was found between school dropouts (children aged 6–17) and EFB after adjusting for other variables, despite a generally higher prevalence amongst those experiencing EFB. It should, however, be noted that we did not disaggregate between children's gender. Further, research in this area is also granted as gender-based discrimination has been reported in Cambodia [124].

These findings align with research from other low and middle-income countries encountering excessive OOPHE [125–128]. Furthermore, despite a decline in direct financing of OOPHE through savings, borrowing, and asset sales, the prospective long-term effects on future revenues, due to loan service demands and asset loss, might pave the way for deteriorated physical and mental health, and possibly diminish human capital [97, 129, 130].

### Decomposition of inequality over time

The 2014–19 substantial increase in EFB inequality was mainly driven by an inequitable rise in a few factors across both thresholds. Urbanization was the primary mitigating factor across. However, population growth in the capital contributed significantly to rising inequality between rural and urban areas.

The protective effect of urbanization on EFB inequality posits ethical questions. It would be dystopian to promote policies to urbanize the entire country or actively relocate population groups. Leaving rural areas behind will necessarily contribute to a social divide, and rapid urbanization in the absence of social security will erode benefits from urbanization, as suggested by the negative effect of the increase in population in Phnom Penh on inequality.

Demographic changes alone do not explain the observed changes. Still, it is alarming that disability-based discrimination may have worsened, as suggested by changes in effects for the share of children under five, and people living with disabilities. Surprisingly, the increasing share of older people had a mitigating contribution. This finding may be due to wealthier households being more likely to have elderly members; either because they can afford healthcare or because they take on the burden of caring for elderly family members within large family networks. More worryingly, this association might be interpreted to suggest that wealthier households are more apt to provide the necessary healthcare for their members to reach older age.

Furthermore, the rapid rise in inequalities in durable goods consumption contributed to exacerbating EFB inequality across thresholds, and so did, to a smaller extent, education spending (but for EFB10 only). Worth noting was a mitigating but non-significant effect of education spending.

For EFB25 only, the rising discrimination towards the wealthy in unspecific loans contributed to rising inequality. Addressing this discrimination by promoting borrowing for illness costs should be cautiously interpreted. Kolesar et al. (2021) explore non-for-profit-commercial credits for healthcare [97]. The challenges in setting up such policies are substantial and should not be uncoupled from questions on their limited social solidarity.

The average OOPHE increase mitigated inequality, suggesting that the poor absorbed most of the rise. Most households did not have any OOPHE. Thus, a marginal increase in the mean technically decreased inequality as the differential between the high and low spenders was reduced. However, the changes in the OOPHE effect almost compensated for this for EFB10 inequality, and worsened EFB25 inequality.

Reducing the share of non-income-financing OOPHE was associated with mitigation of EFB. But as for OOPHE, discrimination in the availability of these coping strategies actually contributed to a deepening of inequality, particularly savings-financed OOPHE.

Unsurprisingly, the increase in the prevalence of illness or injury worsened inequality. However, its strong exacerbating effect on EFB25 inequality suggests that the adverse impact on the less-wealthy has worsened. On the positive side, despite substantial jumps, variations in prevalence activity days lost to illness, hospitalization, and seeking medical healthcare had comparatively modest contributions to increasing inequality. Also, rises in preventive care mitigated inequality substantially.

Improvements in HEF had a notable mitigating effect on inequality, suggesting gains in the effectiveness and equity of the system. However, no significant contribution could be found for coverage changes as this remained stable between 2014–19, at 10.3% of households. Furthermore, HEF-free healthcare in the 12 months decreased. This low coverage contrasts with the official figures for the latest year that we could find, 2017, of ~ 2.9 million people covered, or 18.3% of the population [92]. Worth noting, our estimates put NSSF coverage at 14.92% of households in 2019, close to the officially reported 2.3 million, or 14.4% of the population, in 2021 [27].

## Conclusions

The overall increase in consumption may have contributed to making services more accessible and reducing the FB of the majority of the Cambodian population. However, our findings also show that the increase in material wealth has not benefited every household equitably. The continuous rise in FB, particularly among households in the lowest 40% of the wealth spectrum of the population, shows that the economic gains from peace and political stability of the last decades have yet to be redistributed and translated into the equitable financial burden needed to secure human capital growth. The increasing EFB inequality, the contribution of durable goods, and changes in the effects of the share of people living with disabilities and children under five suggest an urgent need for policy measures to secure social cohesion in Cambodia.

The nationwide extension of the HEF in 2015 marked a significant social policy intervention [92]. Our findings suggest that while HEF has gained in effectiveness and improved access to free healthcare, its impact on reducing FB and inequality is nuanced by its slow expansion. Our findings also illustrate that the concern of misallocation of HEF benefits to the non-poor is, to the most extent, unjustified and that an extension of exemptions and social health assistance through population or condition-specific targeting is a valid policy to reduce financial burden and inequality.

Extending social health protection to the entire population through the NSSF may be part of the solution to tackle inequality. However, the low average income from salaried work, minimum wage (~ US$200), and salary ceilings on contributions make redistributing the last decade’s economic growth unpractical through contributive health insurance only [99].

Following the COVID-19 pandemic, the Cambodian government expanded the IDPoor program in 2020 to include cash transfers to vulnerable families [98, 131]. The expansion of the program, further investments in quality public health services, expansion of the HEF, and removal of non-financial barriers to access healthcare may well contribute in the medium-term to reductions in inequality.

## Appendix 1

### Determinants of inequality in excessive financial burden

#### Results

Appendix Table 11 *provides the results of RIF regression on the Erreygers Concentration index for EFB10 and EFB25 incidence in 2019. Coefficients were rescaled by a 100 factor.*

Inequality in EFB is significantly influenced by where households live. A one percentage point (pp) increase in the share of households living in urban areas would increase the ECI for EFB10, and subsequently decrease inequality by 0.44%, from -11.28 to -11.23. The same variation would reduce inequality in EFB25 by 0.64%. A one pp increase in the share of households in Phnom Penh would increase inequality in EFB10 by 1.23% and 1.31% for EFB25.

Marginal variations in household size would, in general, not significantly influence inequality, except for increases in the number of small households with 1–2 members. A one pp increase in the share of these would reduce inequality by 0.69% inequality in EFB10.

Increasing the share of elderly, people 60 years old and over, among household members by one pp would decrease inequality by 1.21% and 1.34% for EFB10 and EFB25, respectively. It is worth noting that neither the share of children under five years old nor people living with disabilities has significant marginal effects on inequality.

Only marginal variations among household heads with higher levels of education would significantly influence inequality. A one pp increase in this category would increase inequality by 1.29% for EFB10 and 1.44% for EFB25.

Neither social protection marginal changes in coverage through HEF nor NSSF significantly influence inequality. However, an overall increase in OOPHE exemptions of one pp across households would reduce inequality by 1.26% for EFB10 and 1.32% for EFB25.

Not all health conditions had significant marginal effects on inequality. However, an increase of one pp in respiratory infections would increase inequality in EFB10 by 3.21% and in EFB25 by 4.65%. Increases in circular disease prevalence would increase inequality in EFB10 by 2.73% and EFB25 by 1.39%. A similar increase in preventive services awareness or intake would decrease inequality in EFB10 by 2.20% and EFB25 by 2.90%.

Among the variables on health-seeking behavior considered (hospitalization rates, activity days lost, and inpatient days per hospitalization), only hospitalizations significantly influenced inequality. An increase in one pp of hospitalizations increases inequality in EFB10 by 1.52% and 3.27% in EFB25.

Depending on the primary purpose of the loan, this significantly influences inequality in EFB. An increase in the average loan related to illness of 1 INT$ per 100 household members (equivalent to 1 cent INT$ per member per capita) decreases inequality in EFB10 by 2.47%. Unspecified loans significantly influence inequality in EFB25. However, their effect is weaker, with inequality increasing by 0.20%.

Unsurprisingly, marginal variations in total consumption and OOPHE affect inequality distribution most. An increase in EXP one INT$ per 100 household capita would increase inequality in EFB10 by 14.91% and 10.20% in EFB25. However, the same increase in OOPHE would decrease inequality in EFB10 by 63.88% and 63.75% in EFB25.

The source of financing of OOPHE significantly and strongly influences inequality in EFB10 and, in most cases, in EFB25. An increase in one pp in the funding of OOPHE through savings increases inequality in EFB10 by 1.13% and 0.95% in EFB25. The same increase in financing of OOPHE through borrowing increases inequality in EFB10 by 7.84% and in EFB25 by 9.9%. Financing OOPHE through selling assets increases inequality in EFB10 by 10.55% but does not significantly influence EFB25.

#### Discussion

The RIF regression analysis on EFB’s ECI revealed that the increase in urban-dwelling households, small households (1–2 members), the share of elderly in households, preventive services consumption and needs, OOPHE exemptions, average per capita illness loan, and average per capita OOPHE significantly and positively impacted inequality reduction across EFB thresholds. Conversely, residing in Phnom Penh, higher education level of household heads, prevalence of respiratory infectious and circulatory system diseases, number of hospitalized household members, total per capita consumption, and an elevated share of non-income-financed OOPHE from all sources exerted significant negative effects. However, neither circulatory system disease prevalence nor the proportion of selling-assets-financed OOPHE significantly influenced EFB25 inequality.

There is only limited rationale to advise for policy measures promoting small-childless or mono-parental families, particularly as this could adversely affect social capital and the existing social support networks. The same social capital may explain the positive effect of increasing the share of older people. Promoting higher education could also be a recommendation based on the findings. However, the marginal effect of such measures may be statistically perceived in the short term. It is questionable if this will benefit most of the population.

The protective effect of urbanization on mitigating EFB inequality posits ethical questions. It would be dystopian to promote policies to urbanize the entire country or actively relocate population groups. Leaving rural areas behind will necessarily contribute to a social divide, and rapid urbanization in the absence of social security will erode benefits from urbanization, as suggested by the negative effect of the increase in population in Phnom Penh on inequality.

The positive effect of increasing loans or borrowing for illness costs should be cautiously interpreted. Kolesar et al. (2021) explore non-for-profit-commercial credits for healthcare in a recent publication [97]. The challenges in setting up such a system are substantial, regardless of its limited social solidarity. Instead, the positive marginal effect of increasing exemptions per household suggests that fulfilling the strengthening and effective extension of HEFs should be pursued.

Addressing inequality-influencing disease categories like respiratory and circulatory diseases by integrating increased preventive service uptake is needed to tackle inequality. If these interventions would, in addition, reduce hospitalizations, synergic positive effects on inequality could be gained.

Most households did not have any OOPHE. Thus, a marginal increase in the mean technically decreased inequality as the differential between the high and low spenders is reduced. Similarly, a marginal increase in reliance on non-income-financing OOPHE strategies would reduce inequality. However, both average OOPHE and its non-income-finance cannot be considered as these are already disproportionally burdening the poorest households. Rather, our findings show the current effectiveness of coping strategies and the reliance on them by households with less wealth.

### Appendix tables and figures

**﻿Appendix Fig. 9:**
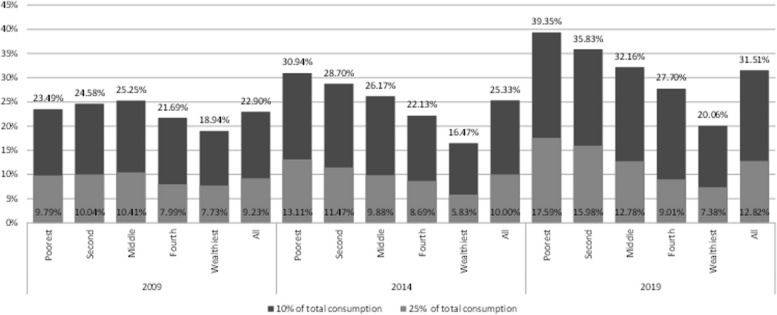
Excessive financial burden incidence among households that reported health needs or healthcare consumption, defined at 10% and 25% of household’s budget by socio-economic quintile and the national level (all households) for households [in % of households]. Source: authors calculations

**Appendix Table 5:** Research questions, corresponding analytical methodology and datasets from the Cambodian Socio-Economic Survey used by the authors. Source: authors calculations ("X": addressed using method)

**Research question**	**Strategy/method**							**Relevant year of dataset**		
	**Socio-economic quantiles and geographic stratification**	**Means, medians, and differences testing**	**Stratification of households according to FB level**	**Concentration indices and differences testing**	**Zero-inflated ordered logistic regression**	**[Recentered influence function regression on concentration indices]**	**Oaxaca-Blinder decomposition of concentration index**	**2009**	**2014**	**2019**
**Primary questions**										
1. How has household healthcare-related financial burden (FB) changed between 2009–19, considering national, quantile, and geographic perspectives?	X	X		X				X	X	X
2. How is FB distributed across Cambodia's population?	X			X				X	X	X
3. What factors (determinants) are associated with FB?			X		X					X
4. Has the inequality in FB shifted over the years?	X			X				X	X	X
5. [What factors (determinants) are associated with marginal changes in FB inequality?]						X				X
6. Which factors explain (compose) the changes in FB inequality over time?							X		X	X
**Secondary questions**										
7. Which strategies do households use to cope with the excessive financial burden of healthcare (EFB)?	X	X			X				X	X
8. How do coping strategies differ between households with EFB and those without?	X	X	X		X				X	X
9. Does the type of health condition and healthcare seeking influence a household's likelihood of facing EFB?			X		X					X
10. Does free healthcare reduce EFB?	X		X		X					X
11. Did the 2015 expansion of HEF reduce FB?	X	X				X	X		X	X

**Appendix Table 6:** Wealth index composition and polychoric dual-component analysis eigenvalues results by year of survey. Source: authors calculations

**Item**	Survey year									
	**2009**				**2014**			**2019**		
	**Coding**	**Eigenvalue**	**Proportion explained**	**Cum. Explanation**	**Eigenvalue**	**Proportion explained**	**Cum. Explanation**	**Eigenvalue**	**Proportion explained**	**Cum. Explanation**
**Housing characteristics**	**-**									
Lighting source	0–5	1.07486	0.032572	0.688424	1.234731	0.037416	0.646724	1.274412	0.038619	0.578102
Public electricity	4									
Generator	5									
Battery	2									
Kerosene lamp	1									
Candle	0									
None	0									
Solar	3									
Cooking fuel source	0–3	1.004372	0.030436	0.71886	1.105785	0.033509	0.680232	1.135133	0.034398	0.6125
Firewood	0									
Charcoal	1									
Gas LPG	2									
Kerosene	1									
Generator	3									
Public electricity	3									
None/does not cook	0									
Other utilities	-									
Garbage collection	0–1	0.119571	0.003623	1.023258	0.150842	0.004571	1.022157	0.174827	0.005298	1.002818
Domestic staff expenses	0–1	-0.908391	-0.027527	1.000000	-0.946645	-0.028686	1.000000	-0.382291	-0.011585	1.000000
Water and sanitation use	-									
Water source access [in wet or all seasons]	0–5	14.135378	0.428345	0.428345	12.435179	0.376824	0.376824	9.717023	0.294455	0.294455
Piped in dwelling or on premises	4									
Public tap	1									
Tubed/piped well or borehole	3									
Protected dug well	2									
Unprotected dug well	1									
Pond, river or stream	0									
Pond, river or stream (pump to the house)	1									
Improved rainwater collection	2									
Unimproved rainwater collection	1									
Water bought (vendor brought water home)	3									
Water bought (collected by houehold)	2									
Bottled water	5									
Other	-									
Sanitation—Toilet [use]	0–4	3.338987	0.101181	0.529526	3.101949	0.093998	0.470822	3.170055	0.096062	0.390518
Flush to sewerage	4									
Flush to septic tank or pit	3									
Flush to elsewhere	2									
Pit latrine with slab	2									
Pit latrine without slab or open pit	1									
Latrine overhanging field or water	1									
Public toilet/pit latrine or shared with others (any type)	1									
Open land	0									
None	-									
Other	-									
Construction materials	-									
Walls material	0–4	1.518923	0.046028	0.575554	1.698308	0.051464	0.522286	1.880073	0.056972	0.447489
Bamboo, Thatch/leaves, Grass	0									
Wood or logs	3									
Plywood	1									
Concrete, brick, stone	4									
Metal sheets	2									
Fibrous cement/Asbestos	2									
Makeshift, mixed materials	0									
Clay/dung with straw	0									
Roof material	0–5	1.367545	0.041441	0.616995	1.456344	0.044132	0.566418	1.602116	0.048549	0.496038
Thatch/leaves/grass	0									
Tiles	4									
Fibrous cement	3									
Metal	2									
Salvaged materials	1									
Mixed expensive materials	5									
Mixed cheap materials	1									
Concrete	5									
Plastic sheet	0									
Other	-									
Floor material	0–5	1.282313	0.038858	0.655853	1.415370	0.042890	0.609308	1.433703	0.043446	0.539484
Earth, clay	0									
Wooden planks	2									
Bamboo strips	1									
Cement/Brick/Stone	3									
Parquet, polished wood	5									
Polished stone, marble	5									
Vinyl	2									
Ceramic tiles	4									
Other	-									
Housing configuration	-									
Rooms per household member equivalent	continuous	0.093270	0.002826	1.026084	0.096036	0.002910	1.025067	0.163895	0.004967	1.007785
Total floor area per household member equivalent	continuous	0.076602	0.002321	1.028406	0.080025	0.002425	1.027492	0.099410	0.003012	1.010797
Ownership of dwelling/Paid rental	0–1	-0.028998	-0.000879	1.027527	0.039413	0.001194	1.028686	0.025986	0.000787	1.011585
**Durable goods, assets**	-									
Home electronics	-									
Radio	0–3	0.889344	0.026950	0.745810	0.980772	0.029720	0.709953	1.066217	0.032310	0.644810
Television	0–3	0.846332	0.025646	0.771456	0.925784	0.028054	0.738007	1.060655	0.032141	0.676951
Video/VCD/DVD player/recorder	0–1	0.794845	0.024086	0.795542	0.868142	0.026307	0.764314	0.913341	0.027677	0.704628
Stereo	0–1	0.779111	0.023609	0.819152	0.792622	0.024019	0.788333	0.845955	0.025635	0.730263
Telephone	0–1	0.714082	0.021639	0.840791	0.740957	0.022453	0.810786	0.821022	0.024879	0.755143
Cell phone	0–3	0.643872	0.019511	0.860302	0.699871	0.021208	0.831994	0.773627	0.023443	0.778586
Satellite dish	0–3	0.548980	0.016636	0.876938	0.672854	0.020390	0.852384	0.742330	0.022495	0.801081
Personal transportation	-									
Bicycle	0–3	0.539708	0.016355	0.893293	0.599118	0.018155	0.870539	0.716684	0.021718	0.822798
Motorcycle	0–3	0.501489	0.015197	0.908489	0.561137	0.017004	0.887543	0.685109	0.020761	0.843559
Car	0–3	0.456968	0.013848	0.922337	0.521719	0.015810	0.903353	0.628520	0.019046	0.862605
Jeep/Van	0–1	0.424821	0.012873	0.935210	0.487098	0.014761	0.918113	0.561167	0.017005	0.879610
Household equipment/appliances	-									
Sewing machine	0–1	0.419604	0.012715	0.947925	0.457098	0.013851	0.931965	0.532325	0.016131	0.895741
Refrigerator	0–3	0.385730	0.011689	0.959614	0.435408	0.013194	0.945159	0.512144	0.015520	0.911261
Air conditioner	0–3	0.316633	0.009595	0.969209	0.391311	0.011858	0.957017	0.464626	0.014080	0.925341
Electric Kitchen/Gas Stove	0–3	0.309418	0.009376	0.978585	0.370532	0.011228	0.968245	0.441942	0.013392	0.938733
Washing machine	0–3	0.286156	0.008671	0.987257	0.342931	0.010392	0.978637	0.408911	0.012391	0.951124
Power sources	-									
Generator	0–1	0.265435	0.008043	0.995300	0.315809	0.009570	0.988207	0.377023	0.011425	0.962549
Batteries	0–3	0.246038	0.007456	1.002756	0.274713	0.008325	0.996532	0.341707	0.010355	0.972904
Furniture	-									
Wardrobe, cabinets	0–3	0.196844	0.005965	1.008721	0.260805	0.007903	1.004435	0.306222	0.009279	0.982183
Computer and printers	-									
Computer (desktop or laptop)	0–3	0.182374	0.005526	1.014247	0.242785	0.007357	1.011792	0.259192	0.007854	0.990037
Agriculture and production	-									
Water pump	0–3	0.177783	0.005387	1.019635	0.191197	0.005794	1.017586	0.246939	0.007483	0.997520

**Appendix Table 7:** Out-of-pocket healthcare expenditure calculation key by financing sources reported in the Cambodian Socio-Economic Survey data

	**Sources of financing**		
**Scenario**	**Source #1**	**Source #2**	**Source #3**
A	1/1 (100.00%)		
B	2/3 (66.67%)	1/3 (33.33%)	
C	4/7 (57.14%)	2/7 (28.57%)	1/7 (14.29%)

**Appendix Table 8:** Descriptive statistics of explanatory variables (observations, percentage of households [%]). Source: authors calculations

	**Survey year**					
	**2009**		**2014**		**2019**	
**Strata**	**Obs**	**%**	**Obs**	**%**	**Obs**	**%**
**Geographic strata**						
Region						
Capital	1,113	8.90%	2,002	11.31%	925	14.62%
Other urban	1,332	9.69%	1,896	11.23%	2,820	23.17%
Other rural	9,525	81.41%	8,192	77.46%	6,330	62.21%
Zone						
Phnom Penh	1,113	8.90%	2,002	11.31%	925	14.62%
Plain	4,993	40.78%	4,127	37.51%	3,200	35.26%
Tonle Sap	3,549	30.22%	3,383	30.62%	2,870	28.90%
Coastal	838	7.30%	600	7.16%	980	6.34%
Plateau/Mountain	1,477	12.80%	1,978	13.40%	2,100	14.88%
Urban/Rural area						
Rural	9,585	82.03%	8,348	78.64%	6,330	62.21%
Urban	2,385	17.97%	3,742	21.36%	3,745	37.79%
**Socio-economic strata**						
Wealth quintile						
Poorest	2,343	20.00%	2,217	20.01%	2,210	20.01%
Second	2,375	20.00%	2,205	20.00%	1,975	20.00%
Middle	2,359	20.00%	2,303	20.00%	1,987	20.00%
Fourth	2,396	20.00%	2,401	20.00%	1,947	20.00%
Wealthiest	2,491	20.00%	2,964	20.00%	1,956	20.00%
**Household (HH) structure**						
Household size [number of members]						
1–2	1,220	10.11%	1,443	11.93%	1,242	12.47%
3–4	4,557	37.96%	5,199	43.06%	4,435	44.61%
5–6	4,179	34.99%	4,037	33.41%	3,321	32.70%
7 and above	2,014	16.95%	1,411	11.60%	1,077	10.22%
Other household characteristics						
Married members under 18 years old	86	0.74%	62	0.53%	74	0.65%
Fully female household	595	4.89%	724	5.98%	492	5.25%
**HH head characteristics**						
Age group						
13–24	478	4.08%	247	2.17%	184	1.66%
25–34	2,435	20.58%	2,284	19.50%	1,590	15.34%
35–44	3,074	25.60%	2,655	22.00%	2,504	24.46%
45–54	2,822	23.48%	3,066	25.08%	2,339	23.28%
55–65	1,889	15.64%	2,300	18.57%	2,064	20.37%
65 and above	1,272	10.61%	1,538	12.67%	1,394	14.89%
Education level						
No class	2,930	25.01%	2,584	22.71%	1,830	17.92%
Primary partial	4,364	36.67%	4,092	35.71%	3,889	37.90%
Primary complete	936	7.78%	941	7.86%	795	8.01%
Secondary lower partial	2,457	20.36%	2,498	20.08%	2,059	20.77%
Secondary lower completed	178	1.43%	234	1.77%	167	1.73%
Secondary upper partial	797	6.44%	922	6.61%	746	7.68%
Secondary upper completed	133	1.06%	404	2.83%	264	2.40%
Higher level	156	1.25%	406	2.42%	319	3.60%
Ethnicity						
Khmer	11,484	95.74%	11,634	96.07%	9,564	95.67%
Cham	306	2.57%	236	2.11%	218	2.19%
Other	180	1.68%	220	1.82%	293	2.14%
Marital status						
Married/in cohabitation	9,486	79.35%	9,361	77.74%	8,120	79.57%
Divorced/Separated	379	3.16%	312	2.53%	223	2.20%
Widowed	1,880	15.57%	2,193	17.93%	1,563	16.46%
Never married or in partnership	225	1.92%	224	1.80%	169	1.77%
Gender, male	9,380	78.44%	9,357	77.71%	8,058	78.50%
Disabled	1,670	14.13%	945	8.00%	963	9.55%
Handicapped	1,670	14.13%	945	8.00%	882	8.76%
**Water and sanitation**						
Access to improved water	5,508	45.40%	6,817	52.55%	7,880	79.73%
Access to improved sanitation	4,419	35.86%	7,399	57.68%	7,859	80.36%
**Social health protection coverage (card holding)**						
Health Equity Fund (HEF) or Priority Access Card	189	1.59%	1,145	10.32%	1,082	10.34%
National Social Security Fund (NSSF)	-	.%	-	.%	1,440	14.92%
**Free healthcare**						
Free healthcare in the last 12 months	579	4.97%	914	8.34%	958	9.58%
HEF free healthcare in the last 12 months	359	3.03%	595	5.43%	387	3.65%
Local poor list healthcare in last 12 months	294	2.49%	463	4.18%	269	2.66%
NSSF free healthcare in the last 12 months	-	.%	-	.%	393	4.20%
Other free healthcare in the last 12 months	291	2.49%	160	1.45%	73	0.71%
Reported free care excl. transportation in the last 30 days [OOPHE = 0]	270	2.31%	307	2.63%	387	3.82%
**Vulnerability**						
Accidents in last 12 months	8,081	67.19%	253	2.15%	415	4.21%
Disability/impairment prevalent	2,769	23.30%	1,706	14.47%	1,702	16.89%
Handicap prevalent					1,587	15.74%
**Liabilities**						
Indebted (unspecified reason)	4,498	37.91%	3,604	31.55%	3,638	34.47%
Indebted because of illness	454	3.83%	277	2.41%	154	1.66%
**Disease prevalence in the last 30 days**						
Healthcare needs or consumption reported	5,706	47.80%	5,889	50.41%	5,787	56.86%
Illness or injury	5,340	44.74%	5,801	49.67%	5,613	55.14%
Long illness (> 1 year)	1,403	11.86%	1,396	11.84%	1,997	19.79%
Non-illness-related care needed	1,622	13.94%	1,779	14.49%	2,274	23.84%
Maternity care (Ante- & postnatal care, delivery)	319	2.72%	168	1.50%	284	2.73%
Prevention (Vit A, deworming, immunization, health checks)	195	1.63%	1,351	10.87%	1,997	21.23%
Other healthcare (non-illness or injury-related)	1,164	10.06%	307	2.49%	72	0.63%
Health condition	5,340	44.74%	5,801	49.67%	5,613	55.14%
Infectious diseases	-	-	3,783	32.34%	3,919	37.85%
Respiratory infectious diseases	-	.%	2,268	18.96%	513	4.88%
Other infectious diseases	-	.%	1,877	16.49%	3,493	33.79%
Chronic diseases					1,933	19.61%
Neoplasms	-	.%	-	.%	104	1.00%
Endocrine, metabolic, and digestive diseases	-	.%	-	.%	604	5.92%
Circular system diseases	-	.%	-	.%	1,062	11.04%
Other chronic diseases	-	.%	-	.%	261	2.60%
Other or undiagnosed diseases	5,340	44.74%	2,346	20.13%	95	0.87%
Injuries/Trauma			25	0.22%	138	1.41%
**Healthcare seeking in the last 30 days**						
Healthcare sought (any providers)	5,407	45.27%	5,781	49.51%	5,731	56.31%
Healthcare for illness/injury sought	4,269	35.45%	5,633	48.31%	5,448	53.36%
Medical healthcare sought	4,238	35.29%	5,174	43.96%	5,351	52.46%
HH members hospitalized	-	.%	423	3.69%	576	5.46%
**Disease impairment in the last 30 days**						
HH members stopping of activities (any)	920	7.74%	599	5.28%	727	7.09%
HH members stopping activities without hospitalization	920	7.74%	411	3.60%	491	4.80%
**Excessive financial burden from healthcare (EFB) incidence in the last 30 days**						
At 10% of consumption, excluding out-of-pocket expenditure	1,306	10.95%	1,436	12.77%	1,850	17.92%
At 25% of consumption, excluding out-of-pocket expenditure	529	4.41%	557	5.04%	742	7.29%
**Catastrophic health expenditure (CHE) in the last 30 days**						
At 40% of national capacity-to-pay [WHO method]	607	5.08%	547	4.90%	784	7.60%
**Coping strategies**						
Coping strategy exhausted between 1–12 months	-	.%	1,636	14.90%	51	0.47%
Coping strategy in the last 12 months	-	.%	1,639	14.92%	211	1.98%
Coping strategy in the last 30 days	-	.%	339	3.14%	174	1.64%
Children 6–14 years old out of schooling prevalent	469	3.93%	303	2.64%	255	2.52%
Children 15–17 years old out of schooling prevalent	1,449	12.19%	1,081	9.47%	793	7.55%
**Out-of-pocket healthcare expenditure (OOPHE) funding sources in the last 30 days**						
Income	-	.%	3,895	32.50%	4,362	42.99%
Savings	-	.%	1,770	15.78%	1,655	15.59%
Borrowing	-	.%	241	2.15%	158	1.62%
Selling of assets and production	-	.%	95	0.84%	34	0.33%
Other unreported	-	.%	165	1.37%	164	1.59%

**Appendix Table 9:** Incidence of excessive financial burden by groups and categories [share of all households]. Source: authors calculations

		**Excessive Financial Burden (EFB)**					
		**Consumption threshold**					
		**10%**			**25%**		
		**Survey year**			**Survey year**		
**Strata**		**2009**	**2014**	**2019**	**2009**	**2014**	**2019**
**All households**		10.95%	12.77%	17.92%	4.41%	5.04%	7.29%
**Geographic strata**							
Region							
Capital		4.30%	3.30%	7.33%	1.86%	1.26%	2.58%
Other urban		7.51%	8.92%	16.53%	2.31%	2.97%	6.20%
Other rural		12.08%	14.71%	20.92%	4.94%	5.89%	8.80%
Zone							
Phnom Penh		4.30%	3.30%	7.33%	1.86%	1.26%	2.58%
Plain		13.57%	15.25%	19.65%	5.34%	6.27%	8.11%
Tonle Sap		9.78%	13.24%	20.06%	4.24%	4.99%	8.18%
Coastal		9.44%	10.08%	15.78%	3.64%	4.67%	6.48%
Plateau/Mountain		10.82%	14.18%	20.98%	4.08%	5.11%	8.58%
Urban/Rural area							
Rural		12.06%	14.53%	20.92%	4.93%	5.82%	8.80%
Urban		5.88%	6.31%	12.97%	2.05%	2.16%	4.80%
**Socio-economic strata**							
Wealth quintile							
Poorest		11.42%	16.42%	24.29%	4.76%	6.96%	10.86%
Second		12.20%	15.60%	21.34%	4.98%	6.24%	9.51%
Middle		12.41%	14.14%	18.55%	5.12%	5.34%	7.37%
Fourth		10.66%	10.82%	15.51%	3.93%	4.25%	5.05%
Wealthiest		8.08%	6.87%	9.90%	3.30%	2.43%	3.65%
**Household (HH) structure**							
Household size [number of members]							
1–2		12.65%	13.24%	20.46%	5.36%	6.11%	9.17%
3–4		10.15%	12.28%	16.42%	4.19%	4.90%	6.94%
5–6		10.37%	12.66%	18.52%	4.10%	4.52%	7.42%
7 and above		12.91%	14.40%	19.41%	5.00%	5.99%	6.10%
Other household characteristics							
Married members under 18 years old	No	10.92%	12.80%	17.88%	4.39%	5.05%	7.30%
	Yes	14.89%	7.40%	23.04%	7.39%	3.21%	5.37%
Fully female household	No	10.87%	12.58%	17.63%	4.38%	4.92%	7.10%
	Yes	12.53%	15.79%	23.20%	5.10%	7.02%	10.62%
**HH head characteristics**							
Age group							
13–24		7.19%	11.92%	15.70%	1.45%	4.14%	7.03%
25–34		8.47%	13.09%	17.97%	3.01%	4.49%	7.84%
35–44		10.12%	11.22%	15.25%	4.39%	4.63%	6.15%
45–54		10.23%	10.20%	16.00%	3.87%	4.53%	6.88%
55–65		12.62%	13.16%	19.22%	5.06%	5.39%	7.10%
65 and above		18.30%	19.63%	23.71%	8.58%	7.27%	9.52%
Education level							
No class		12.52%	15.40%	23.63%	5.20%	6.09%	9.40%
Primary partial		11.67%	14.40%	19.97%	5.09%	5.47%	8.77%
Primary complete		10.55%	12.92%	16.89%	3.30%	6.75%	6.79%
Secondary lower partial		9.88%	10.50%	15.51%	3.51%	4.35%	5.30%
Secondary lower completed		5.54%	11.04%	14.89%	2.01%	2.45%	5.02%
Secondary upper partial		7.31%	7.02%	12.38%	3.25%	2.39%	5.81%
Secondary upper completed		6.94%	7.00%	7.77%	3.52%	2.15%	2.57%
Higher level		5.54%	5.57%	3.99%	0.82%	1.35%	1.19%
Ethnicity							
Khmer		11.18%	12.68%	17.82%	4.56%	5.04%	7.32%
Cham		5.51%	13.63%	22.80%	1.01%	2.18%	8.25%
Other		5.95%	16.72%	17.51%	1.06%	8.52%	4.86%
Marital status							
Married/in cohabitation		11.03%	12.42%	17.69%	4.46%	4.91%	7.24%
Divorced/Separated		9.04%	16.76%	14.16%	3.19%	5.83%	7.81%
Widowed		11.51%	13.63%	20.14%	4.60%	5.42%	7.79%
Never married or in partnership		6.12%	13.72%	12.29%	3.13%	5.82%	4.09%
Gender							
Female		10.88%	13.96%	19.36%	4.28%	5.73%	7.69%
Male		10.97%	12.43%	17.52%	4.45%	4.84%	7.18%
Disabled	No	9.27%	11.88%	16.70%	3.56%	4.69%	6.73%
	Yes	21.15%	23.02%	29.42%	9.58%	9.10%	12.56%
Handicapped	No			16.75%			6.75%
	Yes			30.12%			12.91%
**Water and sanitation**							
Access to improved water	No	11.87%	14.28%	20.96%	4.79%	5.47%	9.49%
	Yes	9.84%	11.41%	17.14%	3.96%	4.65%	6.73%
Access to improved sanitation	No	12.17%	15.08%	22.90%	4.95%	6.33%	9.91%
	Yes	8.75%	11.08%	16.70%	3.45%	4.09%	6.65%
**Social health protection coverage (card holding)**							
Health Equity Fund (HEF) or Priority Access Card	No	10.90%	11.98%	17.53%	4.42%	4.68%	7.07%
	Yes	13.90%	19.65%	21.27%	4.26%	8.20%	9.17%
National Social Security Fund (NSSF)	No			18.09%			7.53%
	Yes			16.95%			5.90%
**Free healthcare**							
Free healthcare in the last 12 months	No	10.95%	12.31%	17.59%	4.47%	4.85%	7.18%
	Yes	10.80%	17.87%	21.00%	3.34%	7.13%	8.34%
HEF free healthcare in the last 12 months	No	10.97%	12.54%	17.74%	4.46%	5.06%	7.17%
	Yes	10.25%	16.79%	22.66%	3.05%	4.77%	10.36%
Local poor list healthcare in last 12 months	No	10.91%	12.49%	17.80%	4.42%	4.85%	7.25%
	Yes	12.35%	19.18%	22.36%	4.06%	9.48%	8.64%
NSSF free healthcare in the last 12 months	No			17.87%			7.30%
	Yes			19.04%			7.01%
Other free healthcare in the last 12 months	No	10.95%	12.79%	17.86%	4.41%	5.00%	7.27%
	Yes	10.91%	11.56%	25.93%	4.74%	7.62%	10.28%
Reported free care excl. transportation in the last 30 days [OOPHE = 0]	No	11.02%	12.87%	18.32%	4.45%	5.07%	7.46%
	Yes	7.87%	9.17%	7.70%	2.78%	4.17%	2.85%
**Vulnerability**							
Accidents in last 12 months	No	11.27%	12.64%	17.76%	4.32%	4.91%	7.22%
	Yes	10.79%	18.52%	21.52%	4.46%	11.08%	8.84%
Disability/impairment prevalent	No	8.21%	11.28%	15.53%	3.06%	4.43%	6.16%
	Yes	19.94%	21.56%	29.65%	8.87%	8.65%	12.81%
Handicap prevalent	No			15.68%			6.22%
	Yes			29.88%			12.98%
**Liabilities**							
Indebted (unspecified reason)	No	9.22%	10.08%	16.41%	3.67%	3.62%	6.65%
	Yes	13.77%	18.61%	20.78%	5.63%	8.13%	8.50%
Indebted because of illness	No	10.31%	11.87%	17.47%	3.92%	4.34%	6.92%
	Yes	27.01%	49.13%	44.64%	16.78%	33.47%	29.00%
**Disease prevalence in the last 30 days**	No						
Health needs or consumption reported	No						
	Yes	22.90%	25.33%	31.51%	9.23%	10.00%	12.82%
Illness or injure	No	0.44%	0.11%	0.31%	0.06%	0.06%	0.15%
	Yes	23.92%	25.60%	32.24%	9.80%	10.09%	13.10%
Long illness (> 1 year)	No	7.43%	8.91%	12.68%	2.77%	3.43%	5.17%
	Yes	37.10%	41.53%	39.14%	16.60%	17.03%	15.87%
Non-illness-related care needs	No	8.99%	9.82%	13.62%	3.61%	3.94%	5.63%
	Yes	23.04%	30.16%	31.63%	9.38%	11.53%	12.57%
Ante- & postnatal care, delivery	No	10.56%	12.49%	17.77%	4.26%	4.87%	7.24%
	Yes	24.71%	31.17%	23.34%	9.82%	16.24%	9.03%
Prevention (Vit A, deworming, immunization & health checks)	No	10.85%	10.57%	14.02%	4.37%	4.30%	5.78%
	Yes	16.52%	30.79%	32.38%	6.84%	11.12%	12.87%
Other healthcare (non-illness or injury-related)	No	9.52%	12.39%	17.78%	3.83%	4.88%	7.25%
	Yes	23.69%	27.82%	38.91%	9.61%	11.43%	13.92%
Health condition	No	0.44%	0.11%	0.31%	0.06%	0.06%	0.15%
	Yes	23.92%	25.60%	32.24%	9.80%	10.09%	13.10%
Infectious diseases (any)	No	10.95%	11.08%	10.32%	4.41%	4.94%	4.17%
	Yes		16.31%	30.40%		5.26%	12.40%
Respiratory infectious diseases	No		13.23%	17.29%		5.35%	7.08%
	Yes		10.82%	30.20%		3.74%	11.26%
Other infectious diseases	No		10.75%	11.30%		4.63%	4.50%
	Yes		23.02%	30.89%		7.15%	12.75%
Chronic diseases							
Neoplasms	No			17.52%			7.11%
	Yes			57.37%			24.57%
Endocrine, metabolic, and digestive diseases	No			16.15%			6.40%
	Yes			46.01%			21.33%
Circular system diseases	No			15.85%			6.54%
	Yes			34.55%			13.32%
Other chronic diseases	No			17.54%			7.12%
	Yes			32.12%			13.41%
Other or undiagnosed diseases	No	0.44%	5.28%	17.72%	0.06%	1.57%	7.20%
	Yes	23.92%	42.51%	39.88%	9.80%	18.83%	16.96%
Injuries & trauma	No		12.66%	17.50%		4.94%	7.06%
	Yes		62.71%	47.47%		50.87%	23.17%
**Disease impairment in the last 30 days**							
HH members stopping of activities (any)	No	7.42%	10.30%	14.55%	2.47%	3.51%	5.32%
	Yes	52.97%	57.16%	62.07%	27.65%	32.55%	33.12%
HH members stopping activities without hospitalization	No	7.42%	11.43%	16.09%	2.47%	4.39%	6.38%
	Yes	52.97%	48.59%	54.17%	27.65%	22.51%	25.20%
**Healthcare seeking in the last 30 days**							
Any healthcare sought	No						
	Yes	24.18%	25.79%	31.82%	9.75%	10.18%	12.94%
Healthcare for illness/injury sought	No	2.49%	0.32%	0.95%	0.75%	0.14%	0.44%
	Yes	26.34%	26.10%	32.75%	11.09%	10.28%	13.27%
Medical healthcare sought	No	1.45%	0.33%	0.77%	0.43%	0.15%	0.25%
	Yes	28.36%	28.63%	33.46%	11.72%	11.28%	13.66%
Household members hospitalized	No		10.74%	14.89%		3.66%	5.24%
	Yes		65.84%	70.31%		41.13%	42.78%
**Excessive financial burden from healthcare (EFB) incidence in the last 30 days**							
At 10% of consumption, excluding out-of-pocket expenditure	No						
	Yes				40.32%	39.48%	40.67%
At 25% of consumption, excluding out-of-pocket expenditure	No	6.83%	8.14%	11.47%			
	Yes						
**Catastrophic health expenditure (CHE) in the last 30 days**							
At 40% of national capacity-to-pay [WHO method]	No	6.22%	8.28%	11.20%	0.52%	0.79%	1.06%
	Yes	99.34%	100.00%	99.69%	77.13%	87.59%	83.07%
**Coping strategies**							
Coping strategy exhausted between 1–12 months	No		11.72%	17.85%		4.59%	7.24%
	Yes		18.78%	31.41%		7.61%	16.41%
Coping strategy in the last 12 months	No		11.72%	17.63%		4.59%	7.12%
	Yes		18.75%	31.98%		7.59%	15.75%
Coping strategy in the last 30 days	No		12.55%	17.68%		4.93%	7.14%
	Yes		19.70%	32.05%		8.62%	16.02%
Children 6–14 years old out of schooling prevalent	No	10.92%	12.74%	17.89%	4.41%	5.03%	7.31%
	Yes	11.52%	13.98%	18.84%	4.51%	5.46%	6.26%
Children 15–17 years old out of schooling prevalent	No	10.58%	12.72%	17.59%	4.19%	4.95%	7.11%
	Yes	13.59%	13.30%	21.89%	6.04%	5.90%	9.41%
**Out-of-pocket healthcare expenditure (OOPHE) funding sources in the last 30 days**							
Income	No		7.71%	9.30%		3.43%	4.11%
	Yes		23.29%	29.35%		8.38%	11.50%
Savings	No		9.74%	14.14%		4.02%	5.74%
	Yes		28.97%	38.35%		10.51%	15.66%
Borrowing	No		11.71%	16.99%		4.21%	6.58%
	Yes		60.86%	74.21%		43.09%	50.02%
Selling of assets and production	No		12.35%	17.69%		4.71%	7.08%
	Yes		61.98%	86.22%		43.82%	71.33%
Other unreported	No		12.40%	17.48%		4.87%	7.02%
	Yes		39.14%	44.67%		17.25%	23.76%

**Appendix Table 10:** Key variables means and concentration indices, annual measures and differences testing for households with healthcare needs or consumption. Source: authors calculations

			**Annual measure**			**Differences**		
			**Survey year**			**Survey years compared**		
**Variable**	**Unit**	**Statistic**	**2009**	**2014**	**2019**	**2014vs2009**	**2019vs2014**	**2019vs2009**
**Excessive financial burden from healthcare (EFB) incidence in the last 30 days**								
At 10% of consumption, excluding out-of-pocket expenditure	Percentage of HHs	Mean	22.90%**	25.33%**	31.51%**	2.44%**	6.18%**	8.61%**
		(Conc.Index)	(-0.037**)	(-0.112**)	(-0.148**)	(-0.075**)	(-0.036*)	(-0.111**)
At 25% of consumption, excluding out-of-pocket expenditure	Percentage of HHs	Mean	9.23%**	10.00%**	12.82%**	0.77%#	2.81%**	3.58%**
		(Conc.Index)	(-0.020**)	(-0.056**)	(-0.089**)	(-0.036**)	(-0.033**)	(-0.069**)
**Consumption**								
Consumption excl. out-of-pocket health expenditure (EXP)	INT$(2011)	Mean	725.48**	984.77**	1,461.34**	259.29**	476.57**	735.86**
		Median	542.78**	802.50**	1,169.30**	259.72**	366.80**	626.52**
		(Conc.Index)	(0.298**)	(0.249**)	(0.269**)	(-0.048**)	(0.019)	(-0.029)
**Out-of-pocket health expenditure (OOPHE)**								
OOPHE as a percentage of EXP	Percent of EXP	Mean	10.61%**	11.72%**	13.49%**	1.11%*	1.77%**	2.88%**
		Median	2.77%	3.20%	4.53%	0.43%	1.33%	1.76%
Out-of-pocket health expenditure (OOPHE), excluding transportation	INT$(2011)	Mean	63.77**	103.23**	161.47**	39.46**	58.24**	97.70**
		Median	15.61	27.14	55.65	11.54	28.50	40.04
		(Conc.Index)	(0.163**)	(0.122*)	(0.126**)	(-0.042#)	(0.005)	(-0.037)
Out-of-pocket health expenditure (OOPHE), including transportation	INT$(2011)	Mean	71.23**	114.89**	176.36**	43.66**	61.47**	105.13**
		Median	19.00	33.93	67.86	14.93	33.93	48.86
		(Conc.Index)	(0.163**)	(0.118*)	(0.128**)	(-0.045*)	(0.010)	(-0.036)
OOPHE by financing source								
Income-financed OOPHE, including transportation	INT$(2011)	Mean		62.30**	117.67**		55.37**	
		Median		10.18	30.54		20.36	
		(Conc.Index)		(0.170**)	(0.196**)		(0.026)	
Savings-financed OOPHE, including transportation	INT$(2011)	Mean		25.83**	40.05**		14.22**	
		Median		0.00	0.00		0.00	
		(Conc.Index)		(0.093)	(0.068)		(-0.025)	
Borrowing-financed OOPHE, including transportation	INT$(2011)	Mean		17.68**	10.67**		-7.01	
		Median		0.00	0.00		0.00	
		(Conc.Index)		(-0.023)	(-0.204**)		(-0.181)	
Selling-assets- and-production-financed OOPHE, including transportation	INT$(2011)	Mean		5.68**	3.31**		-2.36	
		Median		0.00	0.00		0.00	
		(Conc.Index)		(0.003)	(0.010)		(0.006)	
Other-and-unreported-financed OOPHE, including transportation	INT$(2011)	Mean		3.41**	4.66**		1.25	
		Median		0.00	0.00		0.00	
		(Conc.Index)		(0.287)	(-0.248*)		(-0.535*)	
**Social health protection coverage and free healthcare**								
HEF or Priority Access Card holding	Percentage of HHs	Mean	1.95%**	12.82%**	12.07%**	10.87%**	-0.75%	10.12%**
		(Conc.Index)	(-0.031**)	(-0.255**)	(-0.231**)	(-0.224**)	(0.024)	(-0.200**)
Free healthcare in the last 12 months (unspecified)	Percentage of HHs	Mean	5.47%**	11.04%**	12.10%**	5.57%**	1.06%#	6.64%**
		(Conc.Index)	(-0.091**)	(-0.205**)	(-0.117**)	(-0.114**)	(0.088**)	(-0.026)
Free healthcare in the last 12 months (from Health Equity Fund, HEF)	Percentage of HHs	Mean	3.30%**	6.98%**	4.94%**	3.68%**	-2.04%**	1.65%
		(Conc.Index)	(-0.057**)	(-0.137**)	(-0.099**)	(-0.080**)	(0.037**)	(-0.042*)
Free healthcare in the last month (unspecified)	HH members	Mean	0.06**	0.06**	0.08**	0.00	0.02**	0.02**
		(Conc.Index)	(-0.085**)	(-0.232**)	(-0.132**)	(-0.147**)	(0.100#)	(-0.047)
**Liabilities**								
Liabilities (loans), overall, unspecified	INT$(2011)	Mean	703.06**	1,518.35**	6,107.63**	815.29**	4,589.28**	5,404.57**
		(Conc.Index)	(0.243**)	(0.323**)	(0.351**)	(0.080)	(0.028*)	(0.109**)
Liabilities (loans), illness-related	INT$(2011)	Mean	45.54**	89.77**	159.81**	44.23**	70.04*	114.27**
		(Conc.Index)	(-0.181)	(-0.031)	(0.007)	(0.151)	(0.037)	(0.188)
**Healthcare needs in the last 30 days**								
Illness/injury reported	HH members	Mean	1.45**	1.33**	1.28**	-0.13	-0.04**	-0.17
		(Conc.Index)	(0.014)	(0.009**)	(0.000**)	(-0.005*)	(-0.009)	(-0.014*)
Long (chronic—> 1 year) illness	HH members	Mean	0.29**	0.26**	0.40**	-0.03	0.14**	0.10**
		(Conc.Index)	(0.064*)	(0.069)	(0.041)	(0.004)	(-0.028)	(-0.023#)
Non-illness-related care needs	HH members	Mean	0.38**	0.36**	0.52**	-0.03	0.17**	0.14**
		(Conc.Index)	(0.033)	(0.034)	(0.100**)	(0.001)	(0.066**)	(0.067)
**Healthcare seeking in the last 30 days**								
Healthcare visits	Visits	Mean	2.90**	2.34**	2.09**	-0.57**	-0.25	-0.81**
		(Conc.Index)	(0.020)	(0.011**)	(0.020*)	(-0.009#)	(0.009)	(0.000)
Healthcare for illness/injury sought	HH members	Mean	1.15**	1.28**	1.23**	0.14**	-0.05**	0.09**
		(Conc.Index)	(0.013)	(0.008**)	(0.001**)	(-0.005#)	(-0.008)	(-0.012*)
Medical healthcare sought	HH members	Mean	1.09**	1.15**	1.22**	0.05*	0.07**	0.12**
		(Conc.Index)	(0.066**)	(0.021*)	(0.016**)	(-0.045**)	(-0.005)	(-0.049**)
Hospitalizations	HH members	Mean		0.08**	0.11**		0.03**	
		(Conc.Index)		(-0.026*)	(-0.058**)		(-0.032)	
**Disease impairment in the last 30 days**								
Activity days stopped because of illness/injury	Days	Mean	2.42**	1.59**	1.49**	-0.83**	-0.10	-0.93**
		(Conc.Index)	(-0.037*)	(-0.044**)	(-0.099**)	(-0.007)	(-0.055)	(-0.062#)
**Catastrophic health expenditure (CHE) in the last 30 days**								
At 40% of national capacity-to-pay [WHO method]	Percentage of HHs	Mean	10.62%**	9.72%**	13.36%**	-0.90%	3.64%**	2.74%**
		(Conc.Index)	(-0.060**)	(-0.077**)	(-0.113**)	(-0.017#)	(-0.036**)	(-0.053**)

Results of statistical testing are marked according to the test’s *P*-value: **P-value < 0.01; *P-value < 0.05; and, #*P*-value < 0.1

**Appendix Table 11:** Excessive financial burden inequality determinants analysis results using a recentered influence function regression on Erreygers Concentration index on 2019 data, rescaled to 100. Source: authors calculations

	**Excessive Financial Burden (EFB)**					
	**Consumption threshold**					
**Dependent variables**	**10%**			**25%**		
**Result**	**Effect**	**Margins**		**Effect**	**Margins**	
	**coef**	**dydx** ^**a**^	**y + dy**	**coef**	**dydx(a)**	**y + dy**
**Independent variables**						
**Geographic strata**						
Zone [dummy]						
Phnom Penh	-13.83**	1.23%	-11.42	-8.068**	1.31%	-6.25
Plain	0.786	-0.07%	-11.27	-0.650	0.11%	-6.18
Tonle Sap	2.781	-0.25%	-11.25	1.054	-0.17%	-6.16
Coastal	0.638	-0.06%	-11.28	-0.563	0.09%	-6.18
Plateau/Mountain	(omitted)			(omitted)		
Urban areas (base: rural)	4.967*	-0.44%	-11.23	3.970**	-0.64%	-6.13
**Household (HH) structure**						
Household size [number of members] (base: 3–4)						
1–2	7.824*	-0.69%	-11.20	-0.0919	0.01%	-6.18
5–6	0.565	-0.05%	-11.28	0.599	-0.10%	-6.17
7 and above	0.227	-0.02%	-11.28	0.523	-0.08%	-6.17
Children under 5 years old [% of HH members]	-0.106	0.94%	-11.39	-0.0588	0.95%	-6.23
Older persons over 60 years old [% of HH members]	0.136**	-1.21%	-11.15	0.0828*	-1.34%	-6.09
Disabled/impaired members [% of HH members]	-0.114	1.01%	-11.40	-0.109#	1.77%	-6.28
**HH head characteristics**						
Education level (base: no education/class)						
Primary partial	4.715#	-0.42%	-11.23	-1.169	0.19%	-6.19
Primary complete	1.222	-0.11%	-11.27	-4.637#	0.75%	-6.22
Secondary lower partial	3.239	-0.29%	-11.25	-2.329	0.38%	-6.20
Secondary lower completed	-4.142	0.37%	-11.32	-3.151	0.51%	-6.21
Secondary upper partial	-3.439	0.30%	-11.32	-4.302	0.70%	-6.22
Secondary upper completed	-7.178	0.64%	-11.35	-5.323#	0.86%	-6.23
Higher level	-14.52**	1.29%	-11.43	-8.907**	1.44%	-6.26
**Social health protection coverage (card holding) [dummy]**						
Health Equity Fund (HEF) or Priority Access Card	3.194	-0.28%	-11.25	2.472	-0.40%	-6.15
National Social Security Fund (NSSF)	0.448	-0.04%	-11.28	-1.161	0.19%	-6.19
**Healthcare needs in last 30 days [% of HH members = per 100 capita]**						
Long (chronic—> 1 year) illness	-0.178	1.58%	-11.46	-0.0919	1.49%	-6.27
Disease						
Infectious diseases						
Endocrine, metabolic and digestive diseases	-0.212	1.88%	-11.49	-0.261#	4.23%	-6.44
Respiratory infections	-0.362**	3.21%	-11.64	-0.287**	4.65%	-6.46
Other infections	-0.350**	3.10%	-11.63	-0.278**	4.50%	-6.45
Chronic diseases						
Neoplasms	-0.0446	0.40%	-11.33	-0.226	3.66%	-6.40
Circular system diseases	-0.308*	2.73%	-11.59	-0.0856	1.39%	-6.26
Other chronic diseases	-0.544*	4.82%	-11.83	-0.397*	6.43%	-6.57
Injuries/Trauma	-0.691#	6.12%	-11.97	-0.487#	7.89%	-6.66
Non-illness-related care						
Maternal health (Ante- & postnatal care, delivery)	-0.0850	0.75%	-11.37	0.222	-3.60%	-5.95
Prevention (Vit A, deworming, immunization & health checks)	0.248**	-2.20%	-11.03	0.179**	-2.90%	-6.00
**Healthcare seeking in the last 30 days [per 100 households]**						
Hospitalizations [members]	-0.172*	1.52%	-11.45	-0.202*	3.27%	-6.38
Days where activities were stopped because of disease	-0.00156	0.01%	-11.28	-0.00126	0.02%	-6.18
Inpatient days per hospitalization	0.0151	-0.13%	-11.27	0.0155	-0.25%	-6.16
**Free healthcare [per 100 households]**						
Reported free care (exempted persons), excluding transportation in the last 30 days (OOPHE = 0)	0.142**	-1.26%	-11.14	0.0812**	-1.32%	-6.09
**Liabilities [INT$(2011) per 100 capita]**						
Loans (unspecified reason)	-0.0172	0.15%	-11.30	-0.0123*	0.20%	-6.19
Loans for illness	0.279*	-2.47%	-11.00	0.0845	-1.37%	-6.09
**Consumption in the last month (30.4 days)**						
Total consumption excl. OOPHE [Intl. $2011 per 100 capita]	-1.682**	14.91%	-12.96	-0.630**	10.20%	-6.80
**Out-of-pocket health expenditure (OOPHE) in the last month (30.4 days)**						
OOPHE [Intl. $2011 per 100 capita]	7.207**	-63.88%	-4.08	3.936**	-63.75%	-2.24
**Out-of-pocket healthcare expenditure (OOPHE) funding sources in last 30 days [% of OOPHE]**						
Savings-financed OOPHE	-0.127**	1.13%	-11.41	-0.0589*	0.95%	-6.23
Borrowing-financed OOPHE	-0.885**	7.84%	-12.17	-0.617**	9.99%	-6.79
Selling-assets-production-financed OOPHE	-1.190**	10.55%	-12.47	-0.739	11.97%	-6.91
Other-and-unreported-financed OOPHE	-0.331**	2.93%	-11.61	-0.236*	3.82%	-6.41
**Constant**	-1.192	10.57%	-12.47	3.426	-55.49%	-2.75
**Mean RIF (y) ~ = ECI#100**	-11.282			-6.1742		
**Observations**	10,069			10,069		
**R-squared**	0.050			0.045		
Erreygers Concentration index (y)	-0.113**			-0.062**		

Results of statistical testing are marked according to the test’s *P*-value: **P-value < 0.01; *P-value < 0.05; and, #*P*-value < 0.1

^a^dy for categorical variables calculated for a variation of 1% of the category; dy for continuous variables calculated for a variation of 1 unit

**Appendix Table 12:** Erreygers Concentration Index decomposition using recentered influence functions on Excessive Financial Burden (EFB) at 10% of consumption comparing 2014 vs. 2019 years. Source: authors calculations

Excessive Financial Burden	Consumption (10%)								
**Result**	**Means**			**Explained**		**Unexplained**		**Total**	
	**2014**	**2019**	**dx[%]**	**coef**	**% diff**	**coef**	**% diff**	**% diff**	**-dy/dx**
**Independent variables [Units]**									
**Geographic strata**									
Zone									
Phnom Penh	0.113	0.146	29.29%	0.00488**	14.88%	0.00368	11.22%	26.10%	0.37
Plain	0.375	0.353	-6.02%	0.000798#	2.43%	0.00622	18.96%	21.40%	-1.46
Tonle Sap	0.306	0.289	-5.60%	0.000914*	2.79%	0.00332	10.12%	12.91%	-0.95
Coastal	0.072	0.063	-11.48%	0.000232	0.71%	-0.00230	-7.01%	-6.30%	0.23
Plateau/Mountain	0.134	0.149	11.07%	-0.000451	-1.38%	-0.00374	-11.40%	-12.78%	-0.47
Urban areas (base: rural)	0.214	0.378	76.96%	-0.00798*	-24.33%	-0.0150*	-45.73%	-70.06%	-0.37
**Household structure**									
Household size [members]	4.46	4.39	-1.59%	3.96e-05	0.12%	0.0298	90.85%	90.97%	-23.47
Disabled/impaired members [share of household members]	4.82%	5.92%	22.85%	0.00123#	3.75%	0.00904*	27.56%	31.31%	0.56
Children under 5 years old [share of household members]	8.47%	8.35%	-1.47%	-0.000213	-0.65%	0.0172*	52.44%	51.79%	-14.48
Older persons over 60 years old [share of household members]	10.80%	12.69%	17.45%	-0.00302**	-9.21%	-0.00467	-14.24%	-23.45%	-0.55
**Social health protection**									
HEF or Priority Access Card holder [dummy]	0.103	0.103	0.18%	-6.72e-06	-0.02%	-0.00863*	-26.31%	-26.33%	-61.53
**General consumption [INT$(2011) per capita]**									
Housing	47.28	81.33	72.00%	0.00437#	13.32%	-0.00123	-3.75%	9.57%	0.05
Education	7.43	13.17	77.32%	0.00345*	10.52%	-0.00630#	-19.21%	-8.69%	-0.05
Durables	23.43	55.63	137.49%	0.0131**	39.94%	0.00391	11.92%	51.86%	0.15
**Liabilities [INT$(2011) per capita]**									
Unspecific loans	291.31	1,545.00	430.36%	0.00184	5.61%	0.00350*	10.67%	16.28%	0.02
Primarily illness-related loans	13.38	30.68	129.31%	-0.000491#	-1.50%	0.000136	0.41%	-1.08%	0.00
**Healthcare needs in the last 30 days**									
Illness or injury prevalence [share of household members]	16.80%	18.80%	11.87%	0.00535**	16.31%	0.00882	26.89%	43.20%	1.49
Illness prevalent for more than one year (chronic condition) [share of household members]	3.56%	6.35%	78.21%	0.00398*	12.13%	0.00177	5.40%	17.53%	0.09
Injuries/Trauma	0.06%	0.39%	539.20%	0.00121	3.69%	-0.000762*	-2.32%	1.37%	0.00
Non-illness-related care [household members]									
Maternity care (Ante- & postnatal care, delivery)	0.016	0.029	89.38%	0.000152	0.46%	5.04e-05	0.15%	0.62%	0.00
Prevention (Vit A, deworming, immunization & health checks)	0.134	0.261	95.18%	-0.0125**	-38.11%	-0.0134**	-40.85%	-78.96%	-0.34
**Healthcare seeking in the last 30 days**									
Healthcare sought (visits to any providers) [household members]	1.177	1.188	0.87%	2.00e-05	0.06%	0.0114	34.76%	34.82%	16.41
Medical healthcare sought [household members]	0.579	0.692	19.59%	0.00488*	14.88%	-0.00561	-17.10%	-2.23%	-0.05
Household members stopped activities because of illness [dumy]	0.053	0.071	34.28%	0.00255**	7.77%	0.00445#	13.57%	21.34%	0.26
Household members hospitalized [dummy]	0.037	0.055	47.82%	0.00179*	5.46%	0.00198	6.04%	11.49%	0.10
**Free healthcare**									
Reported free care excl. transportation in the last 30 days (OOPHE = 0) [household members]	0.031	0.046	47.89%	-0.00234**	-7.13%	0.00141	4.30%	-2.84%	-0.02
**Out-of-pocket health expenditure (OOPHE)**									
OOPHE [INT$(2011) per capita]	12.11	22.43	85.28%	-0.00701**	-21.37%	0.00539**	16.43%	-4.94%	-0.02
Savings-financed OOPHE [% of OOPHE]	14.03%	12.16%	-13.27%	-0.00236**	-7.20%	0.0161**	49.09%	41.89%	-1.30
Borrowing-financed OOPHE [% of OOPHE]	1.83%	1.18%	-35.77%	-0.00596**	-18.17%	0.00362*	11.04%	-7.13%	0.08
Selling-assets-and-production-financed OOPHE [% of OOPHE]	0.57%	0.20%	-65.56%	-0.00455**	-13.87%	0.00413**	12.59%	-1.28%	0.01
Other-and-unreported-financed OOPHE [% of OOPHE]	1.03%	1.21%	17.13%	0.000560	1.71%	0.00312**	9.51%	11.22%	0.27
**Constant**						-0.0490	-149.39%		
**Dependent variable**									
**Result**				**coef**	**% diff**	**-dy[%]**	**Observations**		
Group 1 (year = 2014)				-0.0799**			(12,090)		
Group 2 (year = 2019)				-0.1130**			(10,075)		
Difference (group 1—group 2)				0.0328**	100.00%	41.05%			
Explained (endowments)				0.00452	13.78%	5.66%			
Unexplained (coefficients and interactions)				0.0283*	86.28%	35.42%			

Results of statistical testing are marked according to the test’s *P*-value: **P-value < 0.01; *P-value < 0.05; and, #*P*-value < 0.1

**Appendix Table 13:** Erreygers Concentration Index decomposition using recentered influence functions on Excessive Financial Burden (EFB) at 25% of consumption comparing 2014 vs. 2019 years. Source: authors calculations

**Excessive Financial Burden**	**Consumption (25%)**								
**Result**	**Means**			**Explained**		**Unexplained**		**Total**	
	**2014**	**2019**	**dx[%]**	**coef**	**% diff**	**coef**	**% diff**	**% diff**	**-dy/dx**
**Independent variables [Units]**									
**Geographic strata**									
Zone									
Phnom Penh	0.113	0.146	29.29%	0.00231**	9.31%	0.00306	12.34%	21.65%	0.50
Plain	0.375	0.353	-6.02%	0.000243	0.98%	0.00740	29.84%	30.82%	-3.43
Tonle Sap	0.306	0.289	-5.60%	0.000489#	1.97%	-6.47e-05	-0.26%	1.71%	-0.20
Coastal	0.072	0.063	-11.48%	9.16e-05	0.37%	-0.00206	-8.31%	-7.94%	0.46
Plateau/Mountain	0.134	0.149	11.07%	-0.000285	-1.15%	-0.00239	-9.64%	-10.79%	-0.65
Urban areas (base: rural)	0.214	0.378	76.96%	-0.00612**	-24.68%	-0.00959*	-38.67%	-63.35%	-0.55
**Household structure**									
Household size [members]	4.46	4.39	-1.59%	0.000326	1.31%	0.0183	73.79%	75.10%	-31.63
Disabled/impaired members [share of household members]	4.82%	5.92%	22.85%	0.00113*	4.56%	0.00755**	30.44%	35.00%	1.03
Children under 5 years old [share of household members]	8.47%	8.35%	-1.47%	-9.50e-05	-0.38%	0.0136**	54.84%	54.46%	-24.85
Older persons over 60 years old [share of household members]	10.80%	12.69%	17.45%	-0.00196**	-7.90%	-0.00436	-17.58%	-25.48%	-0.98
**Social health protection**									
HEF or Priority Access Card holder [dummy]	0.103	0.103	0.18%	-5.28e-06	-0.02%	-0.00551*	-22.22%	-22.24%	-84.85
**General consumption [INT$(2011) per capita]**									
Housing	47.28	81.33	72.00%	0.00220	8.87%	0.00522	21.05%	29.92%	0.28
Education	7.43	13.17	77.32%	0.000924	3.73%	-0.00428#	-17.26%	-13.53%	-0.12
Durables	23.43	55.63	137.49%	0.00592**	23.87%	0.000859	3.46%	27.33%	0.13
**Liabilities [INT$(2011) per capita]**									
Unspecific loans	291.31	1,545.00	430.36%	0.00147	5.93%	0.000544	2.19%	8.12%	0.01
Primarily illness-related loans	13.38	30.68	129.31%	-0.000130	-0.52%	0.000188	0.76%	0.23%	0.00
**Healthcare needs in the last 30 days**									
Illness or injury prevalence [share of household members]	16.80%	18.80%	11.87%	0.00406**	16.37%	0.0293**	118.15%	134.52%	7.60
Illness prevalent for more than one year (chronic condition) [share of household members]	3.56%	6.35%	78.21%	0.000160	0.65%	-0.00508*	-20.48%	-19.84%	-0.17
Injuries/Trauma	0.06%	0.39%	539.20%	0.000670	2.70%	-0.000891**	-3.59%	-0.89%	0.00
Non-illness-related care [household members]									
Maternity care (Ante- & postnatal care, delivery)	0.016	0.029	89.38%	-0.00115*	-4.64%	-0.000730	-2.94%	-7.58%	-0.06
Prevention (Vit A, deworming, immunization & health checks)	0.134	0.261	95.18%	-0.00612**	-24.68%	-0.00753**	-30.36%	-55.04%	-0.39
**Healthcare seeking in the last 30 days**									
Healthcare sought (visits to any providers) [household members]	1.177	1.188	0.87%	0.000115	0.46%	0.00797	32.14%	32.60%	25.09
Medical healthcare sought [household members]	0.579	0.692	19.59%	0.00145	5.85%	-0.00425	-17.14%	-11.29%	-0.39
Household members stopped activities because of illness [dummy]	0.053	0.071	34.28%	0.00163**	6.57%	0.00255	10.28%	16.85%	0.33
Household members hospitalized [dummy]	0.037	0.055	47.82%	0.00227**	9.15%	0.00151	6.09%	15.24%	0.21
**Free healthcare**									
Reported free care excl. transportation in the last 30 days (OOPHE = 0) [household members]	0.031	0.046	47.89%	-0.00133**	-5.36%	6.06e-05	0.24%	-5.12%	-0.07
**Out-of-pocket health expenditure (OOPHE)**									
OOPHE [INT$(2011) per capita]	12.11	22.43	85.28%	-0.00344**	-13.87%	0.00469**	18.91%	5.04%	0.04
Savings-financed OOPHE [% of OOPHE]	14.03%	12.16%	-13.27%	-0.000957*	-3.86%	0.00582#	23.47%	19.61%	-0.99
Borrowing-financed OOPHE [% of OOPHE]	1.83%	1.18%	-35.77%	-0.00417**	-16.81%	0.00201	8.10%	-8.71%	0.16
Selling-assets-and-production-financed OOPHE [% of OOPHE]	0.57%	0.20%	-65.56%	-0.00289**	-11.65%	0.00203*	8.19%	-3.47%	0.04
Other-and-unreported-financed OOPHE [% of OOPHE]	1.03%	1.21%	17.13%	0.000417	1.68%	0.00182*	7.34%	9.02%	0.35
**Constant**						-0.0401	-161.69%		
**Dependent variable**									
**Result**				**coef**	**% diff**	**-dy[%]**	**Observations**		
Group 1 (year = 2014)				-0.0370**			(12,090)		
Group 2 (year = 2019)				-0.0618**			(10,075)		
Difference (group 1—group 2)				0.0248**	100.00%	67.03%			
Explained (endowments)				-0.00279	-11.25%	-7.54%			
Unexplained (coefficients and interactions)				0.0276**	111.29%	74.59%			

Results of statistical testing are marked according to the test’s *P*-value: **P-value < 0.01; *P-value < 0.05; and, #*P*-value < 0.1

## Data Availability

The data used in this study is available from the Cambodian National Institute of Statistics upon request.

## Footnotes

1 On should note that data included in the database are regularly updated and deflators amended retroactively.

## References

[1] WHO. The world health report: health systems financing: the path to universal coverage. Bull World Health Organ. World Health Organization; 2010. Available from: https://iris.who.int/handle/10665/44371. 10.2471/BLT.10.078741 PMC2878164 20539847

[2] Bloom G, Katsuma Y, Rao KD, Makimoto S, Yin JDC, Leung GM. Next steps towards universal health coverage call for global leadership. BMJ. 2019 [cited 2023 Mar 7];365:l2107. Available from: 10.1136/bmj.l2107. 10.1136/bmj.l2107 PMC6533546 31126926

[3] Boerma T, Eozenou P, Evans D, Evans T, Kieny M-P, Wagstaff A. Monitoring Progress towards Universal Health Coverage at Country and Global Levels. PLoS Med. 2014 [cited 2020 Jul 21];11:e1001731. Available from: 10.1371/journal.pmed.1001731. 10.1371/journal.pmed.1001731 PMC4171369 25243899

[4] WHO, World Bank. Tracking Universal Health Coverage: 2021 Global Monitoring Report . First global monitoring report. 2021. Available from: https://www.who.int/publications/i/item/9789240040618.

[5] Verguet S. Defining pathways and trade-offs toward universal health coverage: Comment on “Ethical perspective: Five unacceptable trade-offs on the path to universal health coverage”. Int J Health Policy Manag. 2016;5:445–7. Available from: 10.15171/ijhpm.2016.57. 10.15171/ijhpm.2016.57 PMC4930352 27694674

[6] Norheim OF. Ethical perspective: Five unacceptable trade-offs on the path to universal health coverage. Int J Health Policy Manag. 2015 [cited 2023 Mar 9]. p. 711–4. Available from: https://core.ac.uk/download/pdf/33487883.pdf. 10.15171/ijhpm.2015.184 PMC4629695 26673330

[7] Tangcharoensathien V, Patcharanarumol W, Panichkriangkrai W, Sommanustweechai A. Policy choices for progressive realization of universal health coverage: Comment on “ethical perspective: Five unacceptable trade-offs on the path to universal health coverage”. Int J Health Policy Manag. 2017 [cited 2023 Mar 7];6:107–10. Available from: https://core.ac.uk/download/pdf/217366293.pdf. 10.15171/ijhpm.2016.99 PMC5287926 28812786

[8] Kutzin J. Health financing for universal coverage and health system performance: concepts and implications for policy. Bull World Health Organ. 2013 [cited 2013 Oct 13];91:602–11. Available from: http://www.who.int/entity/bulletin/volumes/91/8/12-113985.pdf. 10.2471/BLT.12.113985 PMC3738310 23940408

[9] Rumbold B, Baker R, Ferraz O, Hawkes S, Krubiner C, Littlejohns P, et al. Universal health coverage, priority setting, and the human right to health. edisciplinas.usp.br. 2017 [cited 2023 Mar 7];390. Available from: https://edisciplinas.usp.br/pluginfile.php/5733670/mod_resource/content/1/RUMBOLD%20Lancet_Universal%20health%20coverage.pdf. 10.1016/S0140-6736(17)30931-5 PMC6728156 28456508

[10] ILO. Social Protection Floors Recommendation, 2012 (No. 202). 2012 [cited 2022 Oct 12]. Available from: https://www.ilo.org/dyn/normlex/en/f?p=NORMLEXPUB:12100:0::NO::P12100_INSTRUMENT_ID:3065524.

[11] Felner E. Closing the ‘escape hatch’: A toolkit to monitor the progressive realization of economic, social, and cultural rights. J Hum Rights Pract. 2009. p. 402–35.

[12] UN - Economic and Social Committee for Asia and Pacific. Towards universal social protection. 2022. Available from: www.unescap.org.

[13] Valentine NB, Koller TS, Hosseinpoor AR. Monitoring health determinants with an equity focus: A key role in addressing social determinants, universal health coverage, and advancing the 2030 sustainable development agenda. Glob Health Action. Taylor and Francis Ltd.; 2016. 10.3402/gha.v9.34247 PMC5165053 27989275

[14] Bonati M, Tognoni G, Sereni F. Inequalities in the universal right to health. Int J Environ Res Public Health. MDPI AG; 2021. p. 1–8. 10.3390/ijerph18062844 PMC8000877 33799530

[15] Hosseinpoor AReza, Bergen Nicole, World Health Organization. Gender E and HR, World Health Organization. Information E and R. National health inequality monitoring : a step-by-step manual. 2017.

[16] WHO, World Bank. Tracking Universal Health Coverage: 2021 Global Monitoring Report. 2022. Available from: https://www.who.int/publications/i/item/9789240040618.

[17] WHO, World Bank. Tracking Universal Health Coverage: 2017 Global Monitoring Report. 2017 [cited 2020 Aug 16]; Available from: https://www.who.int/publications/i/item/9789241513555.

[18] Wagstaff A. Measuring catastrophic medical expenditures: Reflections on three issues. Health Economics (United Kingdom). 2019 [cited 2020 Mar 3];28:765–81. Available from: 10.1002/hec.3881. 10.1002/hec.3881 30986890

[19] Hsu J, Flores G, Evans D, Mills A, Hanson K. Measuring financial protection against catastrophic health expenditures: Methodological challenges for global monitoring. Int J Equity Health. 2018;17. 10.1186/s12939-018-0749-5 PMC5984475 29855334

[20] Sweeney S, Mukora R, Candfield S, Guinness L, Grant AD, Vassall A. Measuring income for catastrophic cost estimates: Limitations and policy implications of current approaches. 2018 [cited 2020 Nov 11];215:7–15. Available from: https://linkinghub.elsevier.com/retrieve/pii/S0277953618304738. 10.1016/j.socscimed.2018.08.041 PMC6171470 30196149

[21] Lu C, Chin B, Li G, Murray CJL. Limitations of methods for measuring out-of-pocket and catastrophic private health expenditures. Bull World Health Organ. 2009 [cited 2020 Jun 14];87:238–44. Available from: http://www.who. 10.2471/BLT.08.054379 PMC2654642 19377721

[22] Njagi P, Arsenijevic J, Groot W. Understanding variations in catastrophic health expenditure, its underlying determinants and impoverishment in Sub-Saharan African countries: A scoping review. Syst Rev. 2018 [cited 2020 Nov 11];7:136. Available from: 10.1186/s13643-018-0799-1. 10.1186/s13643-018-0799-1 PMC6134791 30205846

[23] Hanvoravongchai P, Lo V, Ros Chhun E, Fernandes Antunes A. Cambodian Socio-Economic Survey Analysis Out-of-Pocket Expenditure on Health. Phnom Penh, Cambodia; 2012 Dec.

[24] Jacobs B, De Groot R, Fernandes Antunes A. Financial access to health care for older people in Cambodia: 10-year trends (2004–14) and determinants of catastrophic health expenses. Int J Equity Health. 2016 [cited 2016 Jul 28];15:94. Available from: http://linkinghub.elsevier.com/retrieve/pii/S0140673614613477. 10.1186/s12939-016-0383-z PMC4912821 27316716

[25] Jithitikulchai T, Feldhaus I, Bauhoff S, Nagpal S. Health equity funds as the pathway to universal coverage in Cambodia: care seeking and financial risk protection. Health Policy Plan . 2021 [cited 2021 Nov 24];36:26–34. Available from: https://academic.oup.com/heapol/article/36/1/26/6041060 10.1093/heapol/czaa151 33332527

[26] FernandesAntunes A, Jacobs B, De Groot R, Thin K, Hanvoravongchai P, Flessa S. Equality in financial access to healthcare in Cambodia from 2004 to 2014. Health Policy Plan. 2018;33:906–19. 30165473 10.1093/heapol/czy073

[27] Kaiser AH, Okorafor O, Ekman B, Chhim S, Yem S, Sundewall J. Assessing progress towards universal health coverage in Cambodia: Evidence using survey data from 2009 to 2019. Soc Sci Med. 2023;321:115792. Available from: https://linkinghub.elsevier.com/retrieve/pii/S0277953623001491. 10.1016/j.socscimed.2023.115792 36842307

[28] Kolesar RJ, Bogetoft P, Chea V, Erreygers G, Pheakdey S. Advancing universal health coverage in the COVID-19 era: an assessment of public health services technical efficiency and applied cost allocation in Cambodia. Health Econ Rev. 2022 [cited 2024 Feb 11];12:1–20. Available from: https://healtheconomicsreview.biomedcentral.com/articles/10.1186/s13561-021-00354-8 10.1186/s13561-021-00354-8 PMC8800415 35092482

[29] WHO, World Bank. Tracking Universal Health Coverage: First Global Monitoring Report. Tracking Universal Health Coverage. 2015. Available from: https://apps.who.int/iris/rest/bitstreams/786208/retrieve

[30] WHO, World Bank. Global Monitoring Report on Financial Protection in Health 2019. 2020. Available from: https://apps.who.int/iris/rest/bitstreams/1274733/retrieve.

[31] Fernandes Antunes A, Jacobs B, Jithitikulchai T, Nagpal S, Tong K, Flessa S. Sensitivity analysis and methodological choices on health-related impoverishment estimates in Cambodia, 2009–17. Health Policy Plan . 2022;37:791–807. Available from: https://academic.oup.com/heapol/article/37/6/791/6554426. 10.1093/heapol/czac028 35348681

[32] Verguet S, Woldemariam AT, Durrett WN, Norheim OF, Kruk ME. Is the sustainable development goal target for financial risk protection in health realistic? BMJ Glob Health. 2017;2. 10.1136/bmjgh-2016-000216 PMC5639981 29071127

[33] WHO. SDG Target 3.8 | Achieve universal health coverage, including financial risk protection, access to quality essential health-care services and access to safe, effective, quality and affordable essential medicines and vaccines for all . The Global Health Observatory. [cited 2023 Mar 10]. Available from: https://www.who.int/data/gho/data/themes/topics/indicator-groups/indicator-group-details/GHO/sdg-target-3.8-achieve-universal-health-coverage-(uhc)-including-financial-risk-protection.

[34] Grépin KA, Irwin BR, Sas Trakinsky B. On the measurement of financial protection: An assessment of the usefulness of the Catastrophic Health Expenditure indicator to monitor progress towards Universal Health Coverage. Health Syst Reform . 2020;0:e1744988. 10.1080/23288604.2020.1744988. 10.1080/23288604.2020.1744988 33416439

[35] Zhang Y, Guan Y, Hu D, Vanneste J, Zhu D. The Basic vs. Ability-to-Pay Approach: Evidence From China’s Critical Illness Insurance on Whether Different Measurements of Catastrophic Health Expenditure Matter. Front Public Health . 2021 [cited 2023 Apr 17];9:116. Available from: 10.3389/fpubh.2021.646810/full. 10.3389/fpubh.2021.646810 PMC8044960 33869132

[36] Xu K, Evans DB, Kawabata K, Zeramdini R, Klavus J, Murray CJ. Household catastrophic health expenditure: a multicountry analysis. Lancet. 2003;362:111–7. 12867110 10.1016/S0140-6736(03)13861-5

[37] Koch SF. Catastrophic health payments: Does the equivalence scale matter? Health Policy Plan . 2018 [cited 2018 Sep 10];33:966–73. Available from: http://www.ncbi.nlm.nih.gov/pubmed/30107411. 10.1093/heapol/czy072 30107411

[38] Wagstaff A, Flores G, Hsu J, Smitz MF, Chepynoga K, Buisman LR, et al. Progress on catastrophic health spending in 133 countries: a retrospective observational study. Lancet Glob Health . 2018 [cited 2018 Apr 22];6:e169–79. Available from: http://www.ncbi.nlm.nih.gov/pubmed/29248367. 10.1016/S2214-109X(17)30429-1 29248367

[39] Flores G, Krishnakumar J, O’Donnell O, van Doorslaer E. Coping with health‐care costs: implications for the measurement of catastrophic expenditures and poverty. Health Econ . 2008 [cited 2020 Jul 14];17:1393–412. Available from: 10.1002/hec.1338. 10.1002/hec.1338 18246595

[40] Koch SF, Setshegetso N. Catastrophic health expenditures arising from out-of-pocket payments: Evidence from South African income and expenditure surveys. Hotchkiss D, editor. PLoS One . 2020 [cited 2021 Nov 19];15:e0237217. Available from: 10.1371/journal.pone.0237217. 10.1371/journal.pone.0237217 PMC7418962 32780758

[41] Joe W, Rajpal S. Unravelling the socioeconomic gradient in the incidence of catastrophic health care expenditure: a comment. Health Policy Plan . 2018 [cited 2023 Apr 17];33:699–701. Available from: https://academic.oup.com/heapol/article/33/5/699/4956817. 10.1093/heapol/czy026 29617995

[42] Sas Trakinsky B, Irwin BR, Guéné HJL, Grépin KA. An empirical evaluation of the performance of financial protection indicators for UHC monitoring: Evidence from Burkina Faso. Health Policy Open . 2020 [cited 2023 Apr 17];1:100001. Available from: https://linkinghub.elsevier.com/retrieve/pii/S2590229619300012. 10.1016/j.hpopen.2019.100001 PMC10297743 37383309

[43] O’Donnell O, van Doorslaer E, Wagstaff A, Lindelow M. Analyzing Health Equity Using Household Survey Data . Washington, D.C.: World Bank; 2008. Available from: http://scholar.google.com/scholar?hl=en&btnG=Search&q=intitle:Analyzing+Health+Equity+Using+Household+Survey+Data#5.

[44] Erreygers G, Van Ourti T. Measuring socioeconomic inequality in health, health care and health financing by means of rank-dependent indices: A recipe for good practice. J Health Econ . 2011 [cited 2020 Nov 17];30:685–94. Available from: /pmc/articles/PMC3158909/?report=abstract. 10.1016/j.jhealeco.2011.04.004 PMC3158909 21683462

[45] Atkinson AB. On the measurement of inequality. J Econ Theory. 1970;2:244–63.

[46] Haughton J, Khandker SR. Handbook on Poverty and Inequality . Washington, DC: World Bank; 2009 Mar. Available from: http://hdl.handle.net/10986/11985.

[47] Beja EJr. Human Development Index and Multidimensional Poverty Index: Evidence on their Reliability and Validity. Munich Personal RePEc Archive . 2021 [cited 2021 Nov 16]; Available from: https://mpra.ub.uni-muenchen.de/108501/.

[48] Alkire S, Foster J, Seth S, Santos ME, Roche JM, Ballon P. Multidimensional Poverty Measurement and Analysis. Online edition. Oxford Academic, editor. 2015.

[49] Borga LG, D’Ambrosio C. Social Protection and Multi-dimensional Poverty: Lessons from Ethiopia. Paris: India and Peru; 2020.

[50] Zeng W, Zhao P, Zhao Y, Saddique R. The multidimensional relative poverty of rural older adults in China and the effect of the health poverty alleviation policy. Frontiers in Public Health. 2022. 10.3389/fpubh.2022.793673 PMC9354235 35937214

[51] Pulok MH, van Gool K, Hajizadeh M, Allin S, Hall J. Measuring horizontal inequity in healthcare utilisation: a review of methodological developments and debates. European Journal of Health Economics . 2020 [cited 2023 Mar 1];21:171–80. Available from: 10.1007/s10198-019-01118-2. 10.1007/s10198-019-01118-2 31542840

[52] Barrett CB, Carter MR. The Economics of Poverty Traps and Persistent Poverty: Empirical and Policy Implications. J Dev Stud. 2013 [cited 2024 Jun 27];49:976–90. Available from: 10.1080/00220388.2013.785527.

[53] Liu S, Coyte PC, Fu M, Zhang Q. Measurement and determinants of catastrophic health expenditure among elderly households in China using longitudinal data from the CHARLS. Int J Equity Health . 2021 [cited 2021 Nov 24];20:62. Available from: 10.1186/s12939-020-01336-8. 10.1186/s12939-020-01336-8 PMC7893946 33608014

[54] Wagstaff A, Watanabe N. What difference does the choice of SES make in health inequality measurement? Health Econ . 2003 [cited 2023 Jun 9];12:885–90. Available from: 10.1002/hec.805. 10.1002/hec.805 14508873

[55] Shaukat B, Javed SA, Imran W. Wealth Index as Substitute to Income and Consumption: Assessment of Household Poverty Determinants Using Demographic and Health Survey Data. J Poverty . 2020 [cited 2020 Jun 14];24:24–44. Available from: 10.1080/10875549.2019.1678550.

[56] Rutstein SO. Steps to constructing the new DHS Wealth Index . Available from: https://www.dhsprogram.com/programming/wealth%20index/Steps_to_constructing_the_new_DHS_Wealth_Index.pdf.

[57] Smits J, Steendijk R. The International Wealth Index (IWI). Soc Indic Res. 2015;122:65–85.

[58] Yanagisawa S, Soyano A, Igarashi H, Hang V, Oum S, Ura M. Development of a short version of wealth index and its application in research on maternal health knowledge and behavior. J Int Health. 2012;27:141–9.

[59] Filmer D, Pritchett LH. Estimating Wealth Effects without Expenditure Data-or Tears: An Application to Educational Enrollments in States of India. Demography . 2001 [cited 2020 Nov 11];38:115. Available from: http://www.jstor.org/stable/3088292?origin=crossref. 10.1353/dem.2001.0003 11227840

[60] Howe LD, Hargreaves JR, Huttly SRA. Issues in the construction of wealth indices for the measurement of socio-economic position in low-income countries. Emerg Themes Epidemiol . 2008 [cited 2021 May 31];5:1–14. Available from: https://link.springer.com/articles/10.1186/1742-7622-5-3. 10.1186/1742-7622-5-3 PMC2248177 18234082

[61] Poirier MJP, Grépin KA, Grignon M. Approaches and Alternatives to the Wealth Index to Measure Socioeconomic Status Using Survey Data: A Critical Interpretive Synthesis. Soc Indic Res. 2020 [cited 2021 May 31];148:1–46. Available from: 10.1007/s11205-019-02187-9.

[62] Martel P, Mbofana F, Cousens S. The polychoric dual-component wealth index as an alternative to the DHS index: Addressing the urban bias. J Glob Health . 2021 [cited 2022 Feb 16];11:1–19. Available from: /pmc/articles/PMC7897450/. 10.7189/jogh.11.04003 PMC7897450 33643634

[63] Ward P. Measuring the level and inequality of wealth: An application to China. Review of Income and Wealth . 2014 [cited 2024 Jun 27];60:613–35. Available from: 10.1111/roiw.12063. 10.1111/roiw.12063 PMC4308814 25641989

[64] Ataguba JE. Assessing financial protection in health: Does the choice of poverty line matter? Health Economics (United Kingdom). 2020; 10.1002/hec.4172 PMC7756704 33009711

[65] Ataguba JE. A short note revisiting the concentration index: Does the normalization of the concentration index matter? Health Econ . 2022 [cited 2022 Nov 29];31:1506–12. Available from: 10.1002/hec.4515. 10.1002/hec.4515 PMC9324972 35426194

[66] Kjellsson G, Gerdtham UG, Petrie D. Lies, Damned Lies, and Health Inequality Measurements: Understanding the Value Judgments. Epidemiology. 2015;26:673–80. 26133019 10.1097/EDE.0000000000000319 PMC4521896

[67] Kakwani N, Wagstaff A, Van Doorslaer E. Socioeconomic inequalities in health: Measurement, computation, and statistical inference. J Econom. 1997;77:87–103.

[68] O’Donnell O, O’Neill S, Van Ourti T, Walsh B, O’Donnell O, O’Neill S, et al. conindex: Estimation of concentration indices. Stata Journal . 2016 [cited 2016 Aug 1];16:112–38. Available from: http://www.ncbi.nlm.nih.gov/pubmed/27053927. PMC4819995 27053927

[69] Erreygers G. Correcting the Concentration Index. J Health Econ. 2009;28:504–15. 18367273 10.1016/j.jhealeco.2008.02.003

[70] Contoyannis P, Hurley J, Walli-Attaei M. When the technical is also normative: a critical assessment of measuring health inequalities using the concentration index-based indices. Popul Health Metr . 2022 [cited 2022 Dec 6];20:21. Available from: 10.1186/s12963-022-00299-y. 10.1186/s12963-022-00299-y PMC9713974 36456956

[71] Xu Y, Zhou Y, Pramono A, Liu Y, Jia C. A 25-Year Trend of Catastrophic Health Expenditure and Its Inequality in China: Evidence from Longitudinal Data. Risk Manag Healthc Policy . 2022 [cited 2022 Oct 18];Volume 15:969–81. Available from: https://www.dovepress.com/a-25-year-trend-of-catastrophic-health-expenditure-and-its-inequality--peer-reviewed-fulltext-article-RMHP. 10.2147/RMHP.S358641 PMC9112452 35592442

[72] Quintal C. Evolution of catastrophic health expenditure in a high income country: Incidence versus inequalities. Int J Equity Health . 2019 [cited 2020 Nov 11];18:145. Available from: https://equityhealthj.biomedcentral.com/articles/10.1186/s12939-019-1044-9 10.1186/s12939-019-1044-9 PMC6749702 31533723

[73] Neelsen S, Eozenou PH-V, Smitz M-F, Wang R. The 2022 Update of the Health Equity and Financial Protection Indicators Database . Washington, DC: World Bank; 2022 [cited 2023 Jun 11]. Available from: http://hdl.handle.net/10986/38399.

[74] Kjellsson G, Gerdtham UG. On correcting the concentration index for binary variables. J Health Econ. 2013;32:659–70. 23522656 10.1016/j.jhealeco.2012.10.012

[75] Wagstaff A. Reply to Guido Erreygers and Tom Van Ourti’s comment on ‘The concentration index of a binary outcome revisited’. Health Econ . 2011;20:1166–8. Available from: 10.1002/hec.1753. 10.1002/hec.1753 21674678

[76] Erreygers G. Correcting the Concentration Index: A reply to Wagstaff . J Health Econ. Elsevier; 2009 [cited 2020 Nov 17]. p. 521–4. Available from: https://linkinghub.elsevier.com/retrieve/pii/S0167629608001938

[77] Paul P. The distributive fairness of out-of-pocket healthcare expenditure in the Russian Federation. Int J Health Econ Manag. 2020 [cited 2021 Apr 24];20:13–40. Available from: 10.1007/s10754-019-09268-9. 10.1007/s10754-019-09268-9 PMC7010690 31197528

[78] Akhtar A, Ahmad N, Roy Chowdhury I. Socio-economic inequality in catastrophic health expenditure among households in India: A decomposition analysis. Indian Econ Rev. 2020 [cited 2020 Nov 23];55:1–31. Available from: 10.1007/s41775-020-00093-3.

[79] Mahmoodi Z, Gill P, Qorbani M, Mohammadian Khonsari N, Sheidaei A, Heshmat R, et al. Socioeconomic inequality in different phenotypes of childhood obesity and its determinants in Iran: a Blinder-Oaxaca decomposition method. BMC Public Health. 2022;22. 10.1186/s12889-022-13997-x PMC9392315 35986309

[80] Sun X, Liabsuetrakul T, Xie X, Liu P. Catastrophic health expenditure and impoverishment for type 2 diabetes mellitus patients in a multiethnic province in China using a Blinder-Oaxaca decomposition: A cross-sectional study. Medicine (United States) . 2019 [cited 2022 Oct 17];98. Available from: https://journals.lww.com/md-journal/Fulltext/2019/09270/Catastrophic_health_expenditure_and_impoverishment.96.aspx. 10.1097/MD.0000000000017376 PMC6775392 31574887

[81] Fu X, Sun Q, Sun C, Xu F, He J. Urban-rural differences in catastrophic health expenditure among households with chronic non-communicable disease patients: evidence from China family panel studies. BMC Public Health . 2021 [cited 2022 Oct 18];21:874. Available from: 10.1186/s12889-021-10887-6. 10.1186/s12889-021-10887-6 PMC8101026 33957893

[82] Rahimi E, Hashemi Nazari SS. A detailed explanation and graphical representation of the Blinder-Oaxaca decomposition method with its application in health inequalities. Emerg Themes Epidemiol . 2021 [cited 2022 Oct 18];18:1–15. Available from: 10.1186/s12982-021-00100-9. 10.1186/s12982-021-00100-9 PMC8343972 34362385

[83] Firpo S, Fortin NM, Lemieux T. Unconditional Quantile Regressions. Econometrica . 2009 [cited 2022 Oct 18];77:953–73. Available from: 10.3982/ECTA6822.

[84] Firpo SP, Fortin NM, Lemieux T. Decomposing wage distributions using recentered influence function regressions. Econometrics. 2018;6.

[85] Rios-Avila F. Recentered influence functions (RIFs) in Stata: RIF regression and RIF decomposition. Stata Journal. 2020 [cited 2021 Apr 24];20:51–94. Available from: 10.1177/1536867X20909690.

[86] Heckley G, Gerdtham UG, Kjellsson G. A general method for decomposing the causes of socioeconomic inequality in health. J Health Econ. 2016;48:89–106. 27137844 10.1016/j.jhealeco.2016.03.006

[87] Asif AM, Akbar M. On the decomposition of rank-dependent indicator of socio-economic inequalities in child malnutrition: Some empirical findings. Socioecon Plann Sci. 2021;77.

[88] Asuman D, Ackah CG, Fenny AP, Agyire-Tettey F. Assessing socioeconomic inequalities in the reduction of child stunting in sub-Saharan Africa. Journal of Public Health (Germany). 2020;28:563–73.

[89] Jithitikulchai T. Do Thai Women Earn Less than Men in Thailand? SSRN Electronic Journal. 2016 [cited 2023 Oct 28]; Available from: https://papers.ssrn.com/abstract=2984737.

[90] World Health Organization, World Bank. Tracking Universal Health Coverage 2021 Global Monitoring Report. Geneva; 2021.

[91] World Health Organization. World health statistics 2022: monitoring health for the SDGs . 2022. Available from: http://apps.who.int/bookorders.

[92] Kolesar RJ, Pheakdey S, Jacobs B, Chan N, Yok S, Audibert M. Expanding social health protection in Cambodia: An assessment of the current coverage potential and gaps, and social equity considerations. Int Soc Secur Rev . 2020 [cited 2020 Aug 21];73:35–63. Available from: 10.1111/issr.12227.

[93] Jacobs B, Price N. A comparative study of the effectiveness of pre-identification and passive identification for hospital fee waivers at a rural Cambodian hospital . Studies in HSO&P. 2008. Available from: http://dspace.itg.be/bitstream/handle/10390/2539/2008shso0437.pdf?sequence=2.

[94] Hanson K, Worrall E, Wiseman V. Targeting services towards the poor: a review of targeting mechanisms and their effectiveness. Health, economic development and household poverty: from understanding to action. 2007 [cited 2013 Oct 14];134–154. Available from: http://www.eldis.org/fulltext/targeting_services_hanson.pdf.

[95] Kidd S, Wylde E. Targeting the Poorest: An assessment of the proxy means test methodology . Canberra; 2011. Available from: www.ausaid.gov.au.

[96] Kolesar RJ, Bogetoft P, Chea V, Erreygers G, Pheakdey S. Advancing universal health coverage in the COVID-19 era: an assessment of public health services technical efficiency and applied cost allocation in Cambodia. Health Econ Rev. 2022;12. 10.1186/s13561-021-00354-8 PMC8800415 35092482

[97] Kolesar RJ, Erreygers G, Van Dam W, Chea V, Choeurng T, Leng S. Hardship Financing, Productivity Loss, and the Economic Cost of Illness and Injury in Cambodia . HAL open science. 2021. Available from: https://repository.uantwerpen.be/docstore/d:irua:11882. 10.1186/s12939-023-02016-z PMC10559627 37805483

[98] IMF. Cambodia: Staff Report for the 2022 Article IV Consultation . 2022 Nov. Available from: http://www.imf.org.

[99] Kolesar RJ, Pheakdey S, Jacobs B, Phay S. Decision time: Cost estimations and policy implications to advance Universal Health Coverage in Cambodia. J Policy Model. 2020;

[100] Ir P, Jacobs B, Asante AD, Liverani M, Jan S, Chhim S, et al. Exploring the determinants of distress health financing in Cambodia. Health Policy Plan. 2019 [cited 2020 Jul 8];34:i26–37. Available from: https://academic.oup.com/heapol/article/34/Supplement_1/i26/5603549. 10.1093/heapol/czz006 PMC6807511 31644799

[101] Progress on household drinking water, sanitation and hygiene 2000–2020: Five years into the SDGs . Geneva; Available from: http://apps.who.int/bookorders.

[102] OECD. What are equivalence scales . OECD Project on Income Distribution and Poverty. OECD; 2009 [cited 2022 Jul 28]. Available from: https://www.oecd.org/economy/growth/OECD-Note-EquivalenceScales.pdf.

[103] Anyaegbu G. Using the OECD equivalence scale in taxes and benefits analysis. Economic and Labour Market Review. 2010 [cited 2023 Jul 28];4:49–54. Available from: 10.1057/elmr.2010.9.

[104] StataCorp. Stata Statistical Software: Release 17. College Station: StataCrop LP; 2021.

[105] Cameron AC, Trivedi PK. Microeconometrics Using Stata, Second Edition, Volumes I and II . Routledge; 2022 [cited 2023 Aug 1]. Available from: https://www.routledge.com/Microeconometrics-Using-Stata-Second-Edition-Volumes-I-and-II/Cameron-Trivedi/p/book/9781597183598.

[106] Katz MH. Multivariable Analysis . Multivariable Analysis: A Practical Guide for Clinicians. Cambridge University Press; 2006 [cited 2023 Aug 29]. Available from: https://www.cambridge.org/core/product/identifier/9780511811692/type/book.

[107] Diallo AO, Diop A, Dupuy J-F. Analysis of multinomial counts with joint zero-inflation, with an application to health economics. J Stat Plan Inference . 2018;194:85–105. Available from: https://linkinghub.elsevier.com/retrieve/pii/S0378375817301623.

[108] Kelley ME, Anderson SJ. Zero inflation in ordinal data: Incorporating susceptibility to response through the use of a mixture model. Stat Med. 2008;27:3674–88. 18351711 10.1002/sim.3267 PMC2572084

[109] StataCorp. Stata 17 Base Reference Manual: ‘ziologit’ Zero-inflated ordered logit regression. College Station, TX: Stata Press; 2021.

[110] WHO, Cambodian Ministry of Health. Financial health protection in Cambodia, (2009–16): analysis of data from the Cambodia Socioeconomic Survey. Manila, Philippines; 2019.

[111] Sangar S, Dutt V, Thakur R. Economic burden, impoverishment and coping mechanisms associated with out-of-pocket health expenditure: analysis of rural-urban differentials in India. Journal of Public Health (Germany). 2018 [cited 2020 Nov 11];26:485–94. Available from: 10.1007/s10389-018-0904-x.

[112] le R Booysen F. Urban–rural inequalities in health care delivery in South Africa. Dev South Afr . 2003 [cited 2023 Sep 19];20:659–73. Available from: 10.1080/0376835032000149298.

[113] Ghiasvand H, Abolghasem Gorji H, Maleki M, Hadian M. Catastrophic Health Expenditure Among Iranian Rural and Urban Households, 2013 - 2014. Iran Red Crescent Med J . 2015;17. Available from: http://ircmj.neoscriber.org/en/articles/16757.html. 10.5812/ircmj.30974 PMC4601211 26473081

[114] Banerjee S. Determinants of rural-urban differential in healthcare utilization among the elderly population in India. BMC Public Health . 2021 [cited 2023 Sep 19];21:939. Available from: 10.1186/s12889-021-10773-1. 10.1186/s12889-021-10773-1 PMC8130530 34001026

[115] Kazemi-Karyani A, Woldemichael A, Soofi M, Karami Matin B, Soltani S, Yahyavi Dizaj J. Explaining Socioeconomic Inequality Differences in Catastrophic Health Expenditure Between Urban and Rural Areas of Iran After Health Transformation Plan Implementation. ClinicoEconomics and Outcomes Research . 2020 [cited 2022 Oct 18];Volume 12:669–81. Available from: https://www.dovepress.com/explaining-socioeconomic-inequality-differences-in-catastrophic-health-peer-reviewed-article-CEOR. 10.2147/CEOR.S261520 PMC7666980 33204128

[116] Cai J, Coyte PC, Zhao H. Decomposing the causes of socioeconomic-related health inequality among urban and rural populations in China: A new decomposition approach. Int J Equity Health. 2017;16. 10.1186/s12939-017-0624-9 PMC5516311 28720105

[117] Jiang W, Xu X, Tang S, Xu L, Zhang Y, Elbers C, et al. Inequity in healthcare needs, health service use and financial burden of medical expenditures in China: results from a consecutive household monitoring study in Jiangsu Province. BMC Health Serv Res . 2019 [cited 2022 Oct 21];19:966. Available from: 10.1186/s12913-019-4796-4. 10.1186/s12913-019-4796-4 PMC6916066 31842861

[118] Van Damme W, Van Leemput L, Por I, Hardeman W, Meessen B. Out‐of‐pocket health expenditure and debt in poor households: evidence from Cambodia. Wiley Online Library . 2004 [cited 2023 Mar 7];9:273–80. Available from: 10.1046/j.1365-3156.2003.01194.x. 10.1046/j.1365-3156.2003.01194.x 15040566

[119] Pickett KE, Wilkinson RG. Income inequality and health: A causal review. Soc Sci Med . 2015 [cited 2018 Apr 14];128:316–26. Available from: https://linkinghub.elsevier.com/retrieve/pii/S0277953614008399. 10.1016/j.socscimed.2014.12.031 25577953

[120] Prinja S, Dixit J, Gupta N, Dhankhar A, Kataki AC, Roy PS, et al. Financial toxicity of cancer treatment in India: towards closing the cancer care gap. Front Public Health. 2023;11. 10.3389/fpubh.2023.1065737 PMC10316647 37404274

[121] Longo CJ, Fitch MI, Banfield L, Hanly P, Yabroff KR, Sharp L. Financial toxicity associated with a cancer diagnosis in publicly funded healthcare countries: a systematic review. Supportive Care in Cancer . 2020 [cited 2023 Jun 9];28:4645–65. Available from: 10.1007/s00520-020-05620-9. 10.1007/s00520-020-05620-9 32653957

[122] Donkor A, Atuwo-Ampoh V Della, Yakanu F, Torgbenu E, Ameyaw EK, Kitson-Mills D, et al. Financial toxicity of cancer care in low- and middle-income countries: a systematic review and meta-analysis. Supportive Care in Cancer . 2022 [cited 2023 Jun 9];30:7159–90. Available from: 10.1007/s00520-022-07044-z. 10.1007/s00520-022-07044-z PMC9385791 35467118

[123] Kastor A, Mohanty SK. Disease-specific out-of-pocket and catastrophic health expenditure on hospitalization in India: Do Indian households face distress health financing? 2018 [cited 2023 Sep 20]; Available from: 10.1371/journal.pone.0196106. 10.1371/journal.pone.0196106 PMC5945043 29746481

[124] Tann B. ‘A Daughter is Like a Pot of Fish Paste While a Son is Like Pure Gold’: Gendered Conceptions of ‘Human Dignity’ in Cambodia. Australian Feminist Law Journal . 2023 [cited 2024 Jul 3];49:293–318. Available from: https://www.tandfonline.com/action/journalInformation?journalCode=rfem20.

[125] Pinilla-Roncancio M, Amaya-Lara JL, Cedeño-Ocampo G, Rodríguez-Lesmes P, Sepúlveda C. Catastrophic health-care payments and multidimensional poverty: Are they related? Health Econ . 2023 [cited 2023 Apr 25]; Available from: 10.1002/hec.4684. 10.1002/hec.4684 37020350

[126] Thakur R, Ram B, Sangar S. Household Strategies to Cope with the Economic Cost of Illness in Low and Middle Income Countries: A Review Study. SSRN Electronic Journal . 2019 [cited 2023 Apr 25]; Available from: https://papers.ssrn.com/abstract=3328174.

[127] Sparrow R, Poel E Van, Hadiwidjaja G, Yumna A, Warda N, Suryahadi A. Coping with the economic consequences of ill health in Indonesia. Health Econ . 2014 [cited 2023 Mar 7];23:719–28. Available from: 10.1002/hec.2945. 10.1002/hec.2945 23832776

[128] Murphy A, McGowan C, McKee M, Suhrcke M, Hanson K. Coping with healthcare costs for chronic illness in low-income and middle-income countries: A systematic literature review. BMJ Glob Health: BMJ Publishing Group; 2019. 10.1136/bmjgh-2019-001475 PMC6730576 31543984

[129] Mohd Hassan NZA, Mohd Nor Sham Kunusagaran MSJ, Zaimi NA, Aminuddin F, Ab Rahim FI, Jawahir S, et al. The inequalities and determinants of Households’ Distress Financing on Out-off-Pocket Health expenditure in Malaysia. BMC Public Health. 2022;22. 10.1186/s12889-022-12834-5 PMC8900333 35255884

[130] Joe W. Distressed financing of household out-of-pocket health care payments in India: Incidence and correlates. Health Policy Plan. 2015;30:728–41. 24966294 10.1093/heapol/czu050

[131] De Stefani A, Laws A, Sollaci A. Household Vulnerability to Income Shocks in Emerging and Developing Asia: the Case of Cambodia, Nepal and Vietnam, WP/22/64. 2022.

