# Correction: Revisiting a decade of inequality in healthcare financial burden in Cambodia, 2009–19: trends, determinants and decomposition

**DOI:** 10.1186/s12939-024-02298-x

**Published:** 2024-11-28

**Authors:** Adelio Fernandes Antunes, Theepakorn Jithitikulchai, Juergen Hohmann, Steffen Flessa

**Affiliations:** 1 Department of Health Care Management, University of Greifswald, Greifswald, Germany; 2 SOCIEUX+ EU Expertise on Social Protection, Labour and Employment, Brussels, Belgium; 3 Faculty of Economics, Thammasat University, Bangkok, Thailand; 4 Takemi Program in International Health, Harvard T.H. Chan School of Public Health, Boston, USA; 5 World Bank Group, Washington, DC USA; 6 General Inspectorate of Social Security, Luxembourg, Luxembourg

**Correction: Int J Equity Health 23**, ** 196 (2024)**

10.1186/s12939-024-02257-6

Following publication of the original article [1], the authors reported errors in the table and figure numbering. Appendix Fig. 9 should have been Appendix Fig. 1; Appendix Tables 5, 6, 7, 8, 9, 10, 11, 12 and 13 should have been Appendix Tables 1, 2, 3, 4, 5, 6, 7, 8 and 9.

The caption legends for Fig. 7 and Fig. 8 were updated from “Data labels are provided only for results with p-values < 0.05 (**p ≤ 0.01, *p ≤ 0.05).” to “Data labels are provided only for results with p-values < 0.05 (***p ≤ 0.01, **p ≤ 0.05).”

The original article [1] has been updated.
